# A Pilot Clinical Study of a Biomimetic Suction Patch for Improving Wrinkles, Elasticity, Hydration, and Pigmentation via Enhanced Topical Delivery

**DOI:** 10.1111/jocd.71074

**Published:** 2026-07-15

**Authors:** Seunghoon Choi, Hyung‐ki Park, Jaehwan Ahn, Dong‐Hyun Ko, Jin‐Hyun Kim, Byung Woo Hwang, Seongmin Noh, Wonkyu Hong, Keun Ho Lee, Da Wan Kim

**Affiliations:** ^1^ Department SKKU Advanced Institute of Nanotechnology (SAINT) Sungkyunkwan University (SKKU) Suwon Gyeonggi‐do Republic of Korea; ^2^ Mimetics Co., Ltd Suwon Republic of Korea; ^3^ R&D Center LG Household & Health Care (LG H&H) Seoul Republic of Korea; ^4^ Benjamin Clinic Seoul Republic of Korea; ^5^ Human Clinical Skin Testing Center Seoul Republic of Korea; ^6^ Department of Electronic Engineering Korea National University of Transportation Chungju‐si Chungbuk Republic of Korea

**Keywords:** biomimetic patch, cosmetic dermatology, human evaluation, microstructured patch, pigmentation, pilot clinical study, skin elasticity, skin hydration, suction‐based adhesion, wrinkle reduction

## Abstract

**Background:**

Suction‐based bioinspired patches have emerged as noninvasive platforms for enhancing topical delivery; however, clinical validation of their multidimensional skin benefits remains limited.

**Aims:**

This pilot study evaluated the clinical efficacy of a biomimetic suction‐type microstructured patch combined with a cosmetic ampule for improving wrinkles, elasticity, hydration, and pigmentation.

**Patients/Methods:**

Ten healthy female subjects (aged 31–63 years) participated in a 4‐week split‐face study. The test side received both ampule and suction‐type patch application, while the control side received ampule alone. Wrinkle depth was assessed using Antera 3D, elasticity using Ballistometer (CoR), hydration using Epsilon E100, and pigmentation using DermaVision imaging. Measurements were performed at baseline, 2 weeks, and 4 weeks under controlled environmental conditions.

**Results:**

After 2 weeks, the test side showed significant improvements in wrinkle depth (−10.74% vs. −6.27%), elasticity (+4.11% vs. +2.03%), and hydration (+28.63% vs. +11.24%) compared with control. After 4 weeks, pigmentation decreased by 15.77% in the test group versus 4.99% in control. Intergroup differences were statistically significant (*p* < 0.05). No adverse events were observed.

**Conclusions:**

The suction‐type biomimetic patch significantly enhanced multiple skin parameters compared with ampule application alone. These findings support the potential of structure‐mediated, noninvasive suction interfaces as delivery‐enhancing platforms in cosmetic dermatology.

## 1 Introduction

Aging skin represents not only a biological inevitability but also a growing clinical and aesthetic consideration, with fine wrinkles, dehydration, decreased elasticity, and pigmentation emerging as the most visible manifestations. Although the cosmetic industry offers a wide spectrum of interventions claiming to alleviate these signs of aging, the clinical effectiveness and lasting benefit of many topical products remain uncertain [1, 2, 3]. Conventional formulations such as serums and creams often struggle with insufficient dermal penetration due to the barrier function of the stratum corneum, resulting in suboptimal clinical outcomes [2, 3, 4]. To overcome this, a range of physical enhancement methods including microneedles, lasers, and iontophoresis have been introduced, yet these approaches frequently demand clinical expertise, entail user discomfort, and face barriers to daily usability [2, 5, 6, 7, 8, 9, 10, 11, 12]. Consequently, there is a rising need for noninvasive, user‐friendly technologies that deliver visible skin improvement backed by physiological measurements.

Among emerging strategies, suction‐based adhesion systems inspired by nature particularly the octopus sucker have attracted growing interest [13, 14, 15, 16, 17, 18]. These systems generate localized negative pressure through microscale cavities, allowing passive, reversible, and highly conformal skin contact without mechanical tacks or chemical irritants [13, 14, 19, 20]. Such architectural mechanisms have been proposed to enhance the transdermal absorption of active compounds, redistribute skin tension, and improve hydration retention [19, 20, 21, 22]. Compared to devices requiring heat, electricity, or enzymatic triggers, suction‐type patches offer an energy‐free alternative with high biocompatibility, making them particularly promising for cosmetic applications that demand repeated use and user compliance [13, 14, 16, 23]. Transdermal enhancement technologies have been developed using diverse strategies, including chemical enhancers, energy‐assisted systems, hypobaric approaches, and stratum corneum‐bypassing platforms such as microneedles [22, 24]. Within this broader landscape, the present patch was evaluated as a noninvasive, energy‐free, short‐duration, structure‐assisted topical application platform intended for cosmetic use, rather than as a direct head‐to‐head replacement for invasive or device‐dependent transdermal systems.

While conceptually compelling, clinical evidence supporting the benefits of suction‐type architectures in skin care is still needed. Prior research has largely relied on in vitro or animal skin models to investigate parameters such as adhesion force or drug permeability [13, 14, 15, 16, 17, 19, 22, 23, 25]. These studies have often focused on single endpoints such as moisturization or permeability without evaluating comprehensive improvements in elasticity, pigmentation, or wrinkle reduction [2, 19, 22]. Moreover, most prototypes involve rigid components, external pumps, or non‐scalable fabrication methods, which hinder their translation to daily dermatological or cosmetic use [15, 17, 26, 27]. Therefore, the potential of suction‐induced mechanical modulation to drive clinically meaningful skin improvements remains to be demonstrated in human studies.

In this study, we conducted a 4‐week pilot clinical evaluation to examine the feasibility and preliminary clinical benefits of a pre‐developed biomimetic suction‐type microstructured patch used in combination with a cosmetic ampule under practical daily‐use conditions [19, 20, 22, 28]. Rather than assuming that the observed effects arise exclusively from the suction architecture itself, the present study was designed to assess the integrated patch‐assisted response produced under moist application conditions, where conformal contact, local occlusion, hydration retention, and suction‐associated microstructural interaction may act together. We therefore examined four interrelated skin features—wrinkles, elasticity, hydration, and pigmentation—in a split‐face trial involving healthy adult subjects, using objective clinical instruments including Antera 3D, Ballistometer BLS780, Epsilon E100, and DermaVision.

This work provides human‐centered pilot evidence that a suction‐type microstructured patch can support measurable cosmetic skin improvements under noninvasive and short‐duration application conditions. At the same time, because a flat non‐suction occlusive patch was not included as separate control in the present design, the relative contribution of the suction microstructure could not be fully isolated from nonsuction occlusion or moisture‐retention effects. Prior studies are known as protuberance‐containing suction structures can enhance negative‐pressure generation, adhesion, and delivery‐related performance compared with hole or flat controls [22]. Accordingly, the present study is intended to provide preliminary clinical and mechanistic context for a structure‐assisted patch platform.

## 2 Materials and Methods

### 2.1 Suction‐Type Patch Design and Mechanism

**FIGURE 1:**
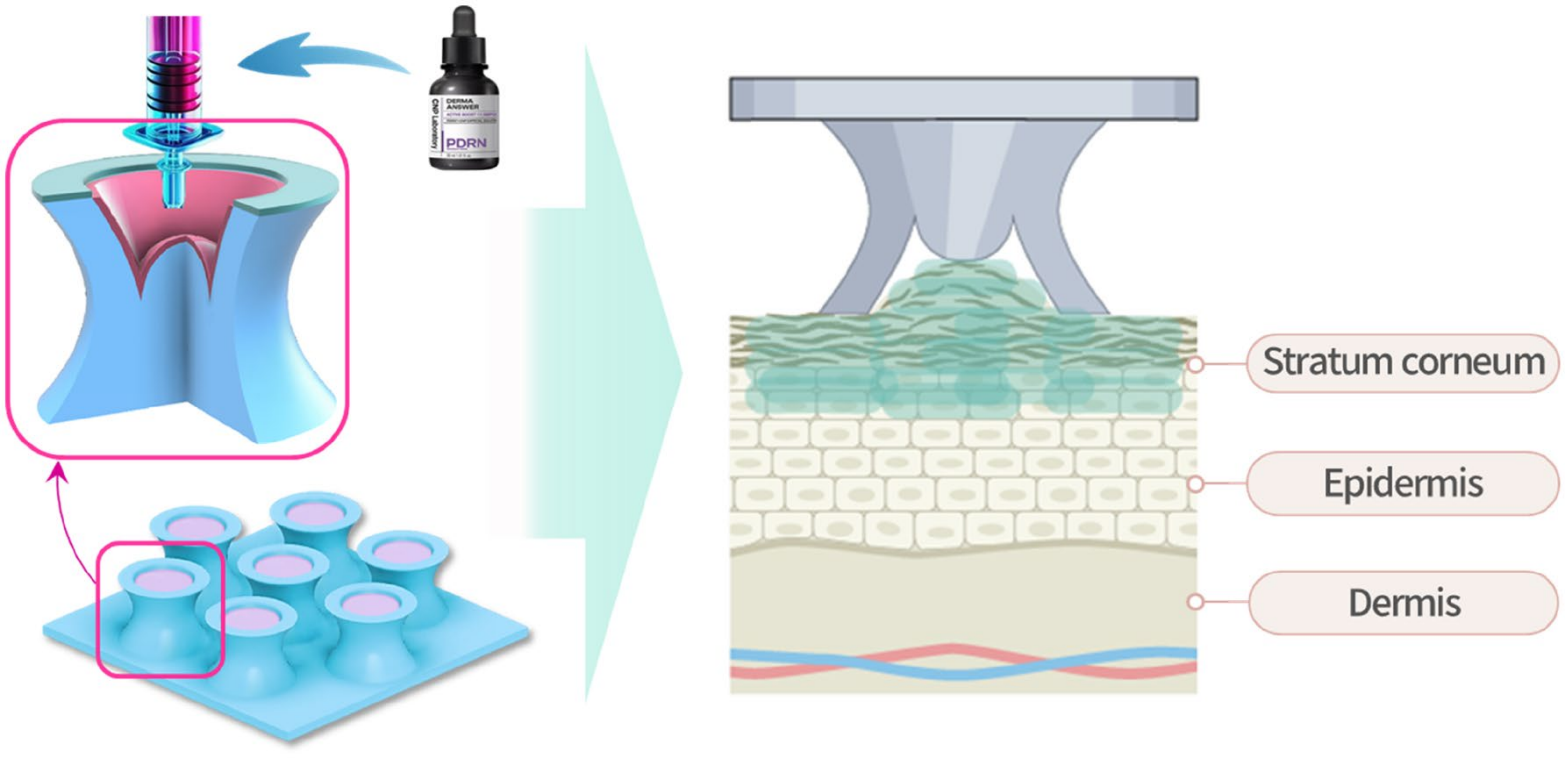
Schematic illustration of biomimetic suction‐type microstructured patch and its skin interface.

The adhesive patch evaluated in this study adopts a suction‐type biomimetic architecture inspired by octopus suckers. The suction‐type microstructured patch was fabricated by Mimetics Co. Ltd. (Suwon, Korea) using medical‐grade liquid silicone via an injection molding process. The silicone material, supplied by Dow Chemical, is widely used in skin‐contact biomedical and wearable applications due to its established biocompatibility and chemical stability [29, 30]. Further details on the patch fabrication process and material preparation are provided in the Supporting Information. As shown in Figure 1, the surface of the patch features an array of microscale cavities that can generate suction‐associated interfacial behavior when applied to the skin. This structural design allows the patch to adhere conformally to curved facial surfaces without the need for chemical adhesives or external energy input. The central microcavity is engineered to deform slightly upon contact, which may increase intimate skin contact and local retention under moist application conditions.

Under practical cosmetic use conditions, the patch is interpreted as a structure‐assisted interface in which Suction‐associated fixation and occlusive sealing act together through the octopus‐inspired microcavity architecture. These coupled functions enhance skin contact and formulation residence, leading to hydration retention and cupping stimulation at the skin interface. Because the present study did not include a flat non‐suction occlusive patch as a separate control, the individual contribution of the suction microstructure could not be directly separated from the occlusive function in the current design. Importantly, the patch operates under fully passive conditions without relying on heat, current, or penetration, making it suitable for repeated daily use in sensitive skin areas such as the periorbital region.

### 2.2 Study Design and Protocol

A prospective, single‐center, split‐face exploratory study was conducted to evaluate the dermatological efficacy of the suction‐type microstructured patch in combination with a functional cosmeceutical ampule. A commercially available cosmetic ampule, CNP Derma Answer Active Boost Ampule, was used in this study together with the suction‐type microstructured patch. The ampule was provided by the study sponsor and was applied according to the study protocol under the same daily‐use conditions on both sides of the face. To improve material transparency and to allow readers to better understand the experimental system, the full ingredient composition of the ampule is provided in the Supporting Information. Ten healthy adult female volunteers (aged 31–63 years, mean age 49.8) with visible periorbital wrinkles were enrolled. All participants provided written informed consent, and the study protocol was approved by the Institutional Review Board (IRB) of Human&P Human Clinical Trial Center (IRB No. HD‐IRB‐P24‐0043), in accordance with the Declaration of Helsinki.

Each subject's face was vertically divided into two symmetrical halves. The test side received both the microstructured patch and the ampule, while the control side received ampule application alone. Subjects were instructed to apply both the ampule and the patch once daily in the evening for a period of 4 weeks. The patch was applied for 15 min and then removed. Clinical assessments were performed at baseline, after 2 weeks, and after 4 weeks of use, depending on the evaluation endpoint: wrinkles, elasticity, and hydration were assessed after 2 weeks, while pigmentation was evaluated after 4 weeks.

Participants were instructed not to introduce any new skincare products, medications, or aesthetic procedures during the study period. All evaluations were performed in a controlled environment (22°C ± 2°C, 50% ± 5% RH), and subjects were given a 30‐min acclimatization period prior to each measurement session. No adverse events or significant skin irritation were reported during the study period.

### 2.3 Adhesion Performance Evaluation

To assess the adhesion performance of the suction‐type microstructured patch, a custom‐built equipment (Adhesion tester, Neo‐Plus, Daejeon, Korea) equipped with a suction cup fixture was employed. Microstructured patch and flat‐surfaced control sample were prepared and applied to a skin‐mimicking silicone substrate under both dry and wet conditions. Detachment forces were measured in the normal direction using suction cups of defined diameter (3 mm^2^), and the peak adhesion stress values were recorded. This method enabled standardized comparison of adhesion strength between biomimetic microcavity designs and nonstructured controls, thereby validating the suction‐induced attachment mechanism prior to clinical evaluation.

### 2.4 Clinical Imaging and Measurement Instruments

To objectively evaluate the physiological effects of the patch application, four distinct noninvasive clinical instruments were employed, each dedicated to a specific skin parameter. Skin wrinkles were assessed using Antera 3D (Miravex Ltd., Ireland), which captures high‐resolution 3D surface images to quantify wrinkle depth and skin texture. Skin elasticity was measured using Ballistometer BLS780 (Dia‐Stron Ltd., United Kingdom), which calculates the Coefficient of Restitution (CoR) based on the skin's mechanical rebound response. Skin hydration was evaluated using Epsilon E100 (Biox Systems Ltd., England), a capacitance‐based sensor that provides average dielectric constant (ε) values corresponding to moisture content. Lastly, pigmentation was analyzed using DermaVision (Opto Biomed Co. Ltd., Republic of Korea), which utilizes cross‐polarized, parallel‐polarized, and ultraviolet imaging to visualize melanin distribution across superficial and deeper skin layers.

Each measurement was conducted under standardized environmental conditions (22°C ± 2°C, 50% ± 5% RH), with subjects undergoing a 30‐min acclimatization period prior to evaluation. All instruments were calibrated prior to use, and images were captured from consistent facial regions (periorbital or cheek) to enable intra‐individual comparisons before and after treatment. Detailed descriptions of each instrument, imaging modalities, and representative results (Figures S1–S3) are provided in the Supporting Information.

### 2.5 Statistical Analysis

In addition to the original significance testing, paired between‐condition differences in change scores were additionally summarized for each endpoint to improve interpretation of the split‐face pilot study results. Because each subject served as her own internal control, the incremental effect of patch‐assisted application was quantified as the paired difference between the improvement observed on the test side and that observed on the control side. These paired differences were further reported with effect sizes (Cohen's dz) and 95% confidence intervals (CIs).

## 3 Result and Discussion

To assess the efficacy of the suction‐type microstructured patch under practical use conditions, we conducted a 4‐week split‐face clinical study involving 10 adult female participants. In this design, the same cosmetic ampule formulation was applied to both sides of the face, while the suction‐type microstructured patch was additionally applied only to the test side. This arrangement allowed intra‐individual comparison of the patch‐assisted condition against the ampule‐only condition while reducing intersubject biological variability. Using standardized imaging, sensor‐based measurements, and statistical analyses, we evaluated changes in four major dermatologic domains: skin wrinkles, elasticity, hydration, and pigmentation. Accordingly, the present comparison should be interpreted as reflecting the performance of the tested patch–ampule system under the defined study conditions, rather than as a formulation‐independent effect directly generalizable to all topical products. In addition, although the present data support patch‐assisted enhancement under moist application conditions, the current design did not fully resolve the relative contributions of micro‐suction, passive occlusion, and prolonged interfacial retention within the tested system. This section presents the endpoint‐specific findings of the split‐face clinical comparison across four dermatologic domains—wrinkles, elasticity, hydration, and pigmentation—supported by quantitative measurements and representative imaging data. Within the present study design, these results are interpreted as reflecting the observed performance of the tested patch–ampule system under defined moist application conditions, rather than as direct proof of any single isolated mechanism. The integrated structure‐assisted patch effect is defined here as the combined action of occlusive sealing and octopus‐inspired suction‐associated fixation, which jointly promotes hydration retention and cupping stimulation under moist application conditions, although their individual magnitudes were not separately quantified in the present design.

**FIGURE 2:**
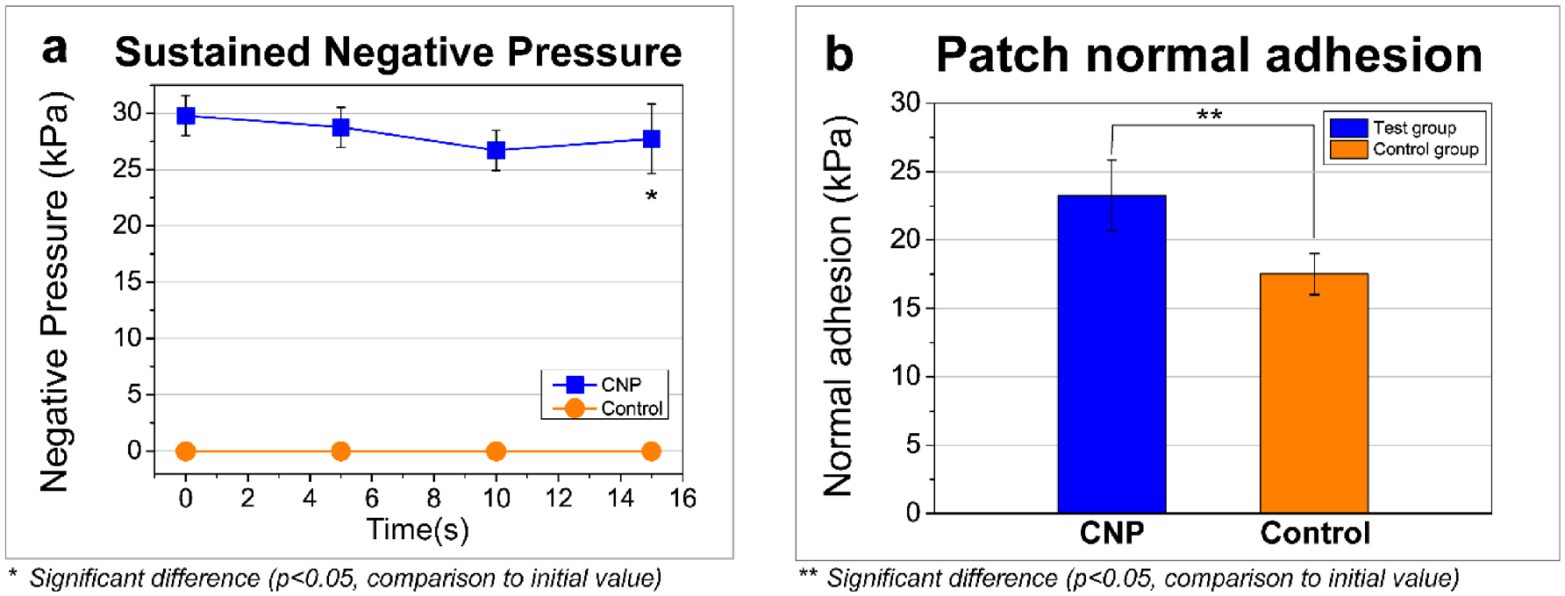
Direct measurement of sustained negative pressure generation and normal adhesion behavior of the microstructured suction‐type patch. (a) Time‐dependent negative pressure generated by the microcavities upon contact under ampule‐treated (CNP) and dry (control) conditions. The negative pressure was continuously monitored and maintained over a 15‐min application period without external vacuum assistance. (b) Quantitative comparison of normal adhesion strength between ampule‐treated (CNP) and dry (control) conditions.

**FIGURE 3:**
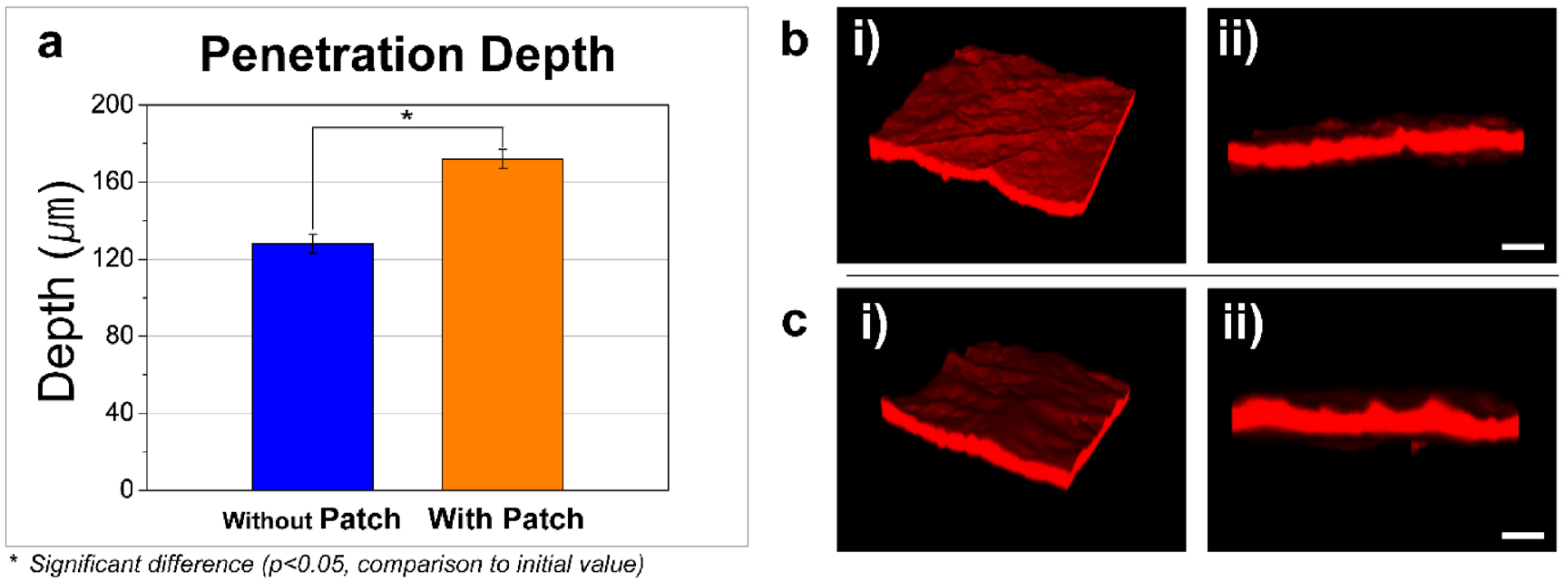
In vivo confocal analysis of penetration depth with and without the suction‐type patch. (a) Quantitative comparison of penetration depth extracted from confocal z‐stack images for skin treated without the patch (Without Patch) and with the suction‐type patch (With Patch). A significant increase in penetration depth was observed upon patch application (*p* < 0.05). (b) Representative confocal images of skin treated without the patch (W/O Patch): (i) three‐dimensional confocal fluorescence reconstruction and (ii) corresponding cross‐sectional (XZ) view showing the depth distribution of the fluorescent tracer.(c) Representative confocal images of skin treated with the suction‐type patch (With Patch): (i) three‐dimensional confocal fluorescence reconstruction and (ii) corresponding cross‐sectional (XZ) view showing the depth distribution of the fluorescent tracer. Scale bars: 200 μm. Data are presented as mean ± SD.

**FIGURE 4:**
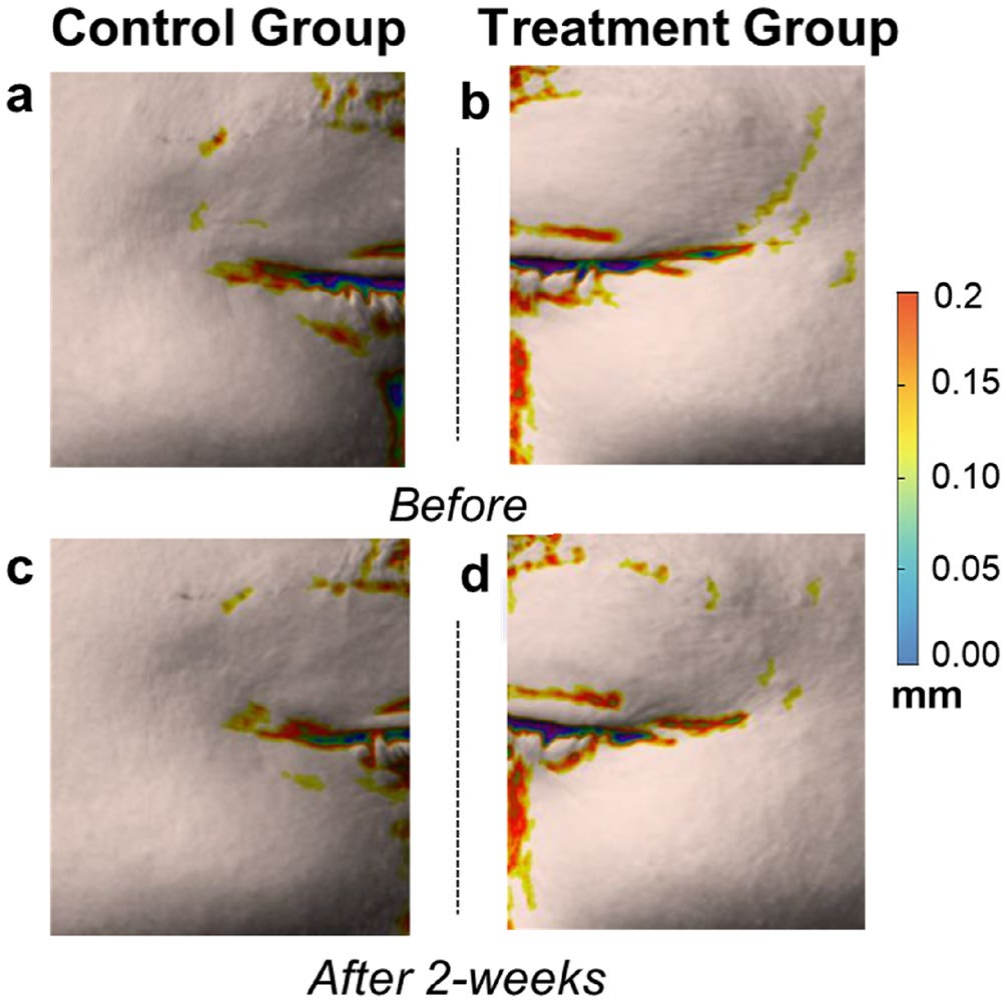
Representative Antera 3D images showing changes in wrinkle depth in the eye area: (a, c) control group before and after 2 weeks, and (b, d) treatment group before and after 2 weeks. Warmer colors indicate deeper wrinkles.

**FIGURE 5:**
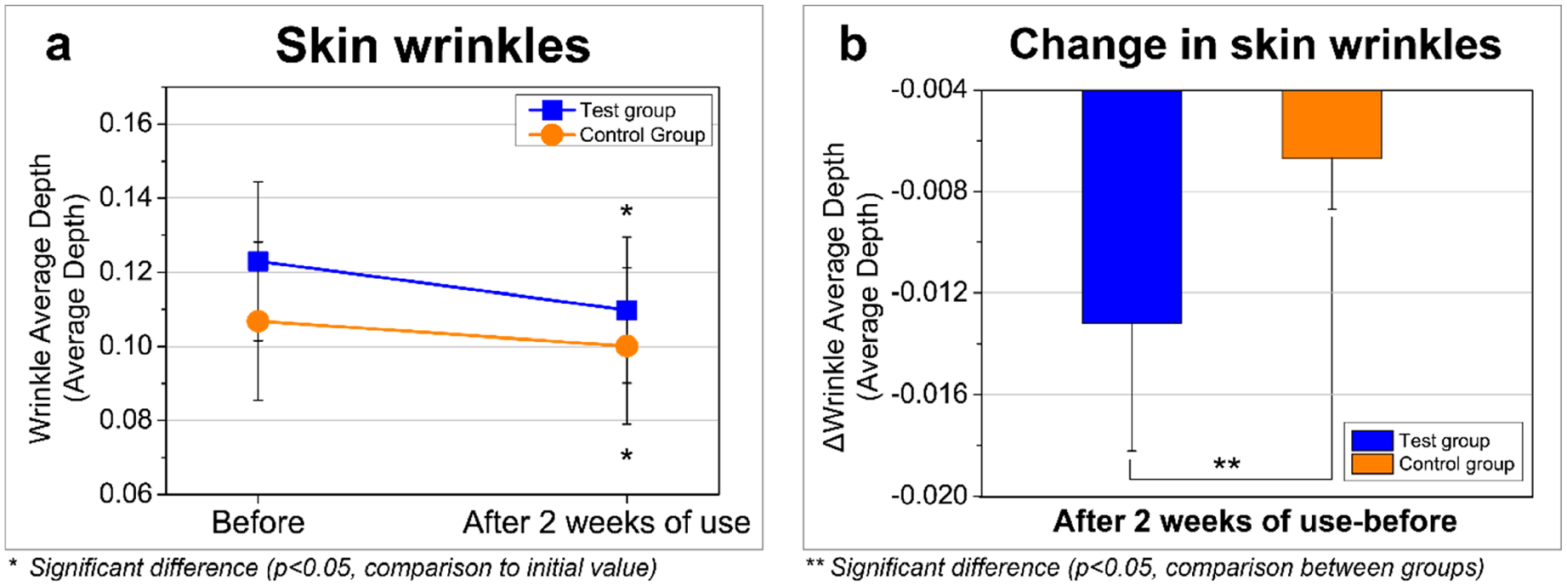
Quantitative evaluation of wrinkle depth in the eye area over 2 weeks: (a) line graph comparing average wrinkle depth between test and control groups before and after application, and (b) bar graph showing Δ (delta) wrinkle depth change, with statistically significant improvement observed in the test group (*p < 0.05, **p < 0.05).

**FIGURE 6:**
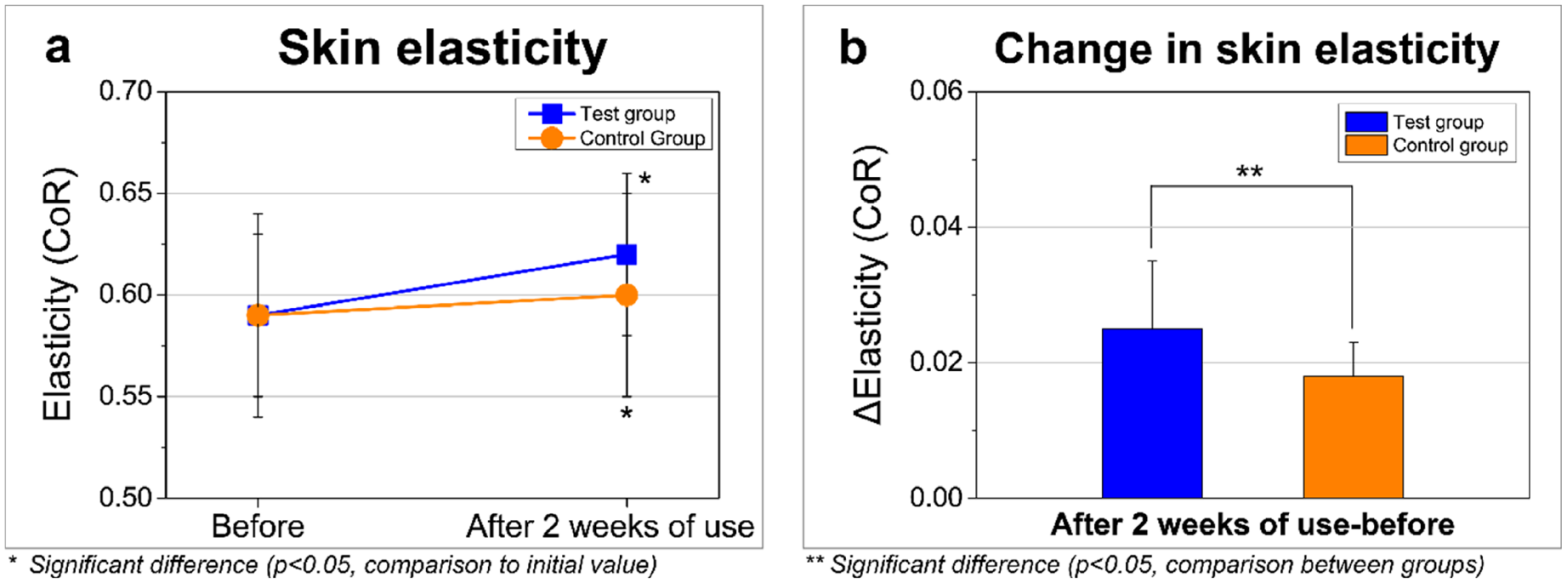
Assessment of skin elasticity using CoR (Coefficient of Restitution): (a) comparison of elasticity values between test and control groups before and after 2 weeks of use, and (b) ΔCoR analysis showing statistically significant improvement in the test group (*p < 0.05, **p < 0.05).

**FIGURE 7:**
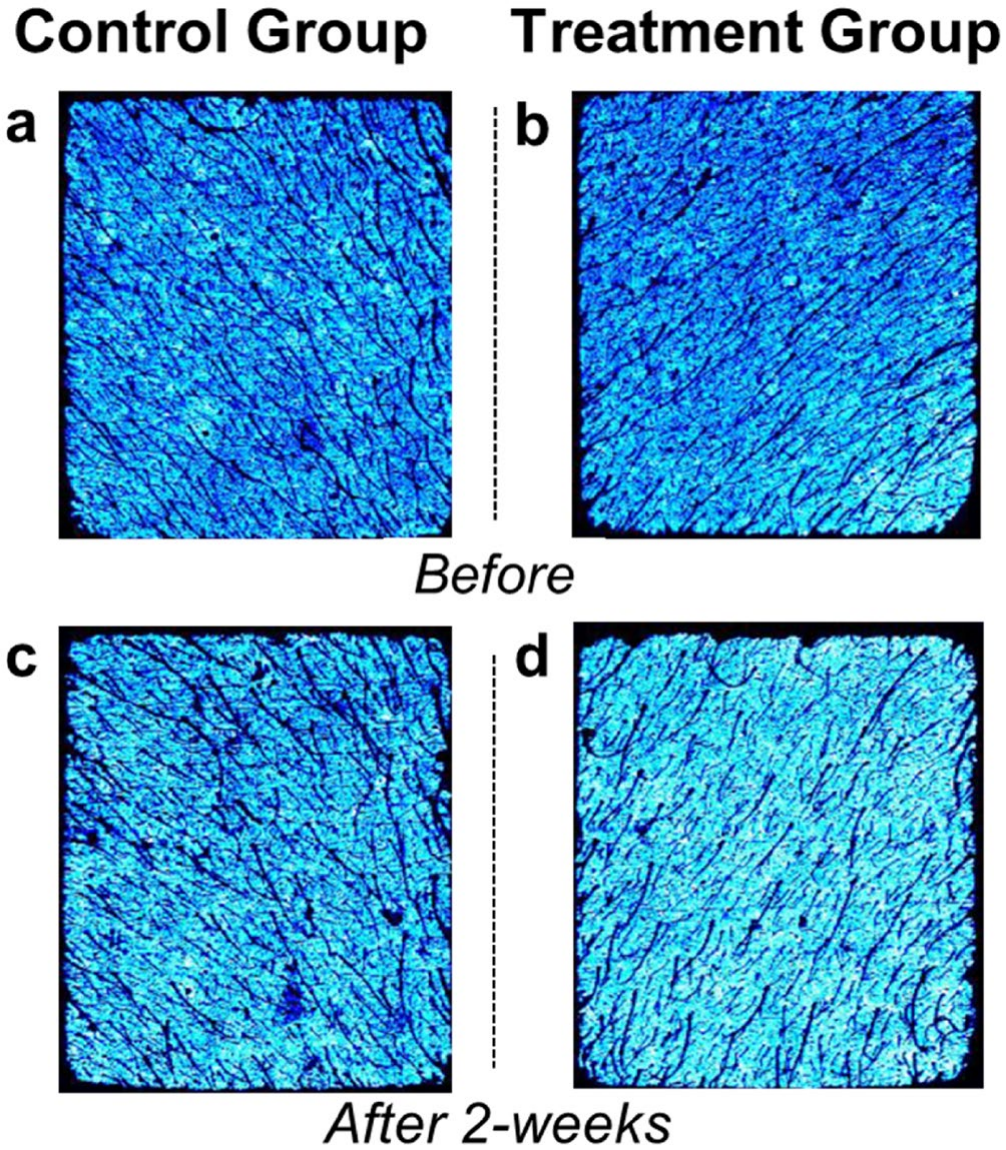
Representative Epsilon E100 hydration images of the cheek area: (a, b) before and (c, d) after 2 weeks of product use, showing visual enhancement in moisture retention in the treatment group compared to the control group.

**FIGURE 8:**
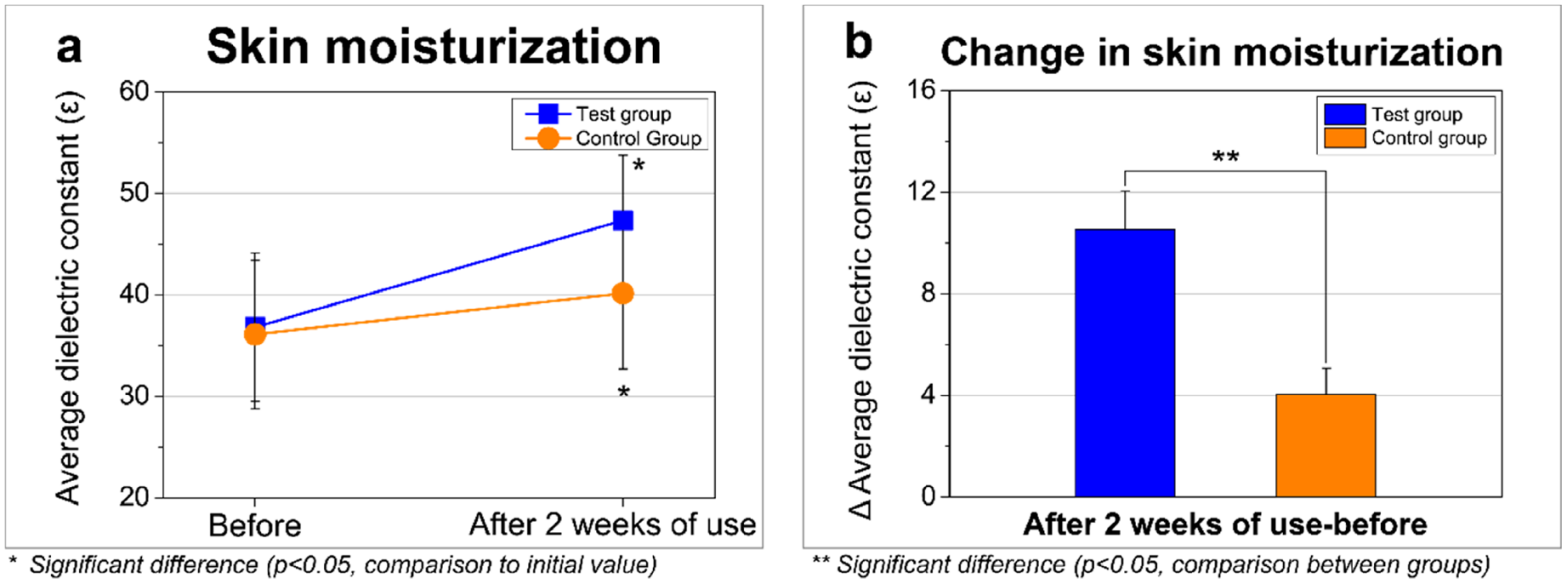
Quantitative assessment of skin hydration using Epsilon E100: (a) change in average dielectric constant (ε) before and after 2 weeks of product use; (b) comparison of hydration improvement (Δε) between treatment and control groups (*p < 0.05, **p < 0.05).

**FIGURE 9:**
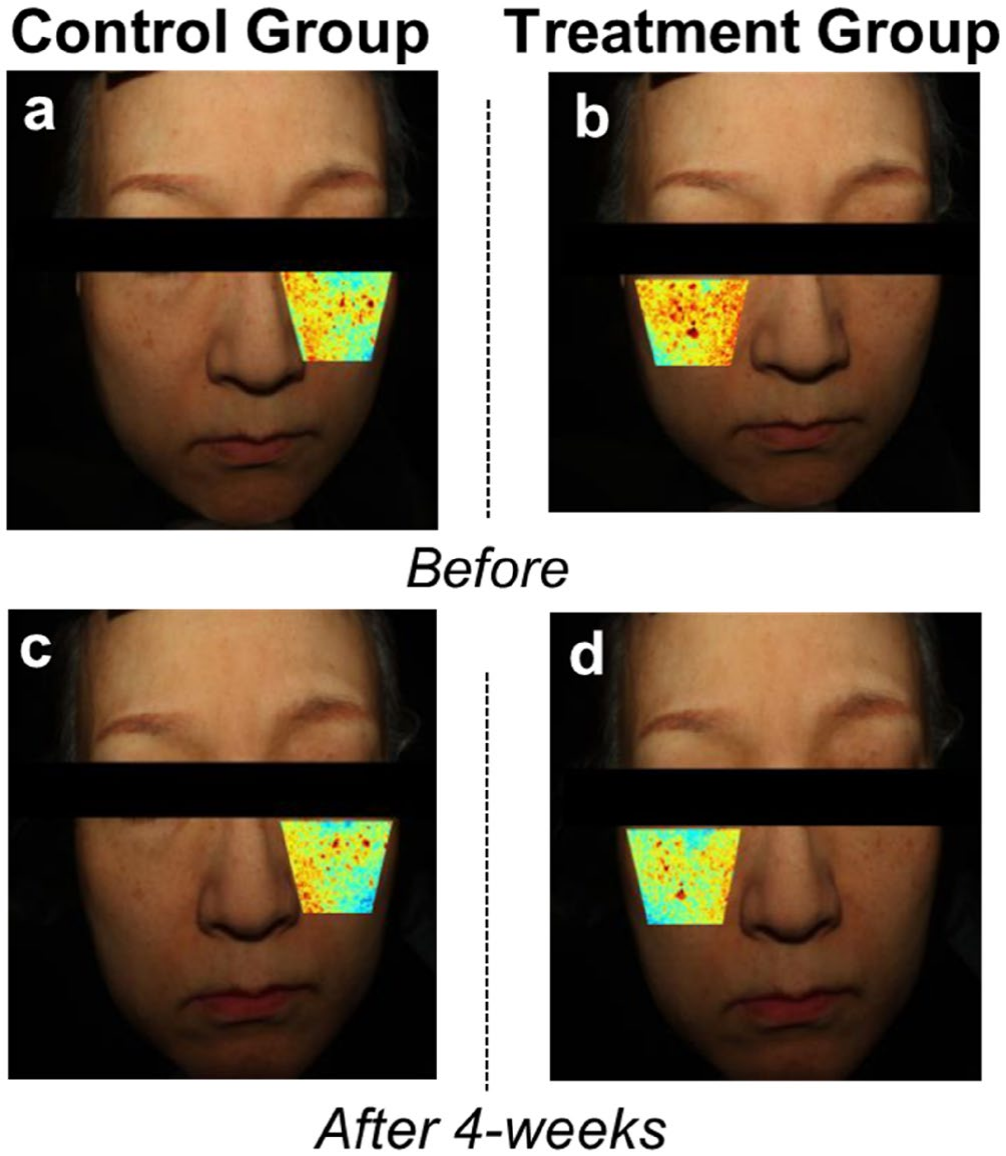
DermaVision‐based melanin distribution maps of four representative subjects: (a, c) control group before and after 4‐week treatment; (b, d) treatment group before and after 4‐week application of suction‐type patch and ampule.

**FIGURE 10:**
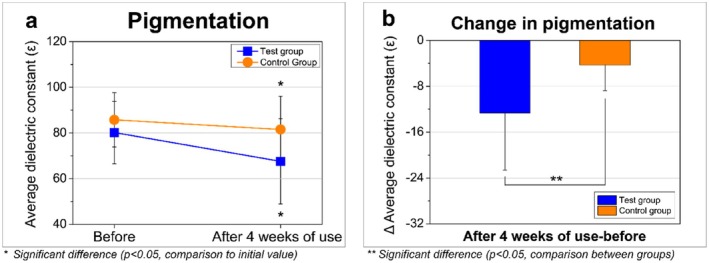
Quantitative assessment of melanin color change (%): (a) average pigmentation levels before and after 4‐week application, and (b) ΔMelanin color (%) comparison between test and control groups, showing significant improvement in the treatment group (*p < 0.05, **p < 0.05).

Each subsection combines visual documentation (Figures 2, 3, 4, 5, 6, 7, 8, 9, 10) and numerical analysis (Tables 1, 2, 3, 4) to summarize the measurable differences observed between the patch‐assisted condition and ampule application alone. Where relevant, mechanistic implications are discussed in light of the current design limitations, particularly the absence of a flat non‐suction occlusive patch control. Within this framework, occlusive sealing and suction‐associated fixation are treated as coupled functions of the octopus‐inspired microcavity architecture that jointly support the observed patch‐assisted performance, although the individual magnitude of each contribution was not separately quantified in the present clinical design.

**TABLE 1:** Wrinkle depth values (mean value ± standard deviation).

	Test group (Mean wrinkle depth)	Control group (Mean wrinkle depth)
Baseline	0.1230 ± 0.0214	0.1068 ± 0.0213
After 2 weeks	0.1098 ± 0.0197	0.1001 ± 0.0211
Intragroup significance^a^ (baseline vs. after 2 weeks)	0.005	0.005
Intragroup significance^b^	< 0.001	
Improvement (%) (baseline vs. after 2 weeks)	10.74%	6.27%
Relative improvement	71.40%	

^a^ *p* value: significant probability, Wilcoxon signed rank test (*p* < 0.05, comparison to initial value).

^b^ *p* value: significant probability, Generalized Estimating Equations (*p* < 0.05, comparison between groups).

### 3.1 Mechanistic Interpretation of Suction‐Associated Microstructures Under Wet Contact Conditions

The present findings should be interpreted within the context of a wet, patch‐assisted interfacial system rather than as a direct isolation study of suction alone. In the current split‐face design, the comparison was performed between ampule alone and ampule plus microstructured patch. Accordingly, the observed differences reflect the integrated effect of patch‐assisted application, which may include conformal contact, local occlusion, hydration retention, prolonged residence of the applied formulation, and suction‐associated interfacial mechanics.

From a mechanistic standpoint, prior studies on octopus‐inspired suction cups under wet contact conditions have described adhesion as arising from suction‐associated and capillary contributions generated during preload‐dependent deformation and vacuum establishment within the chamber [20]. In this context, the theoretical total adhesion under wet contact can be expressed as follows [20]:

$$\sigma_{t,\mathrm{wet}}=\left[\frac{P_{0}\pi D_{\text{in}}^{\prime \prime 2}}{4}+\frac{\pi \left(D_{\text{out}}^{\prime \prime 2}-D_{\text{in}}^{\prime \prime 2}+4r^{2}\right)}{4}\cdot \gamma \left(\frac{\cos\vartheta_{1}+\cos\vartheta_{2}}{h}\right)+l\cdot \gamma \right]\cdot n$$

where $P_{0}$ is atmospheric pressure (∼101.3 kPa), $D_{\text{in}}^{\prime \prime }$ is the inner diameter of the contact surface when a vacuum is established in the inner chamber, $D_{\text{out}}^{\prime \prime }$ is the outer diameter of the contact surface when a vacuum is established in the inner chamber, r is the radius of the dome in contact with the substrate, γ is the surface tension of water, $\theta_{1}$ and $\theta_{2}$ are the contact angles on the adhesive and engaged substrate, h is the liquid gap, and l is the outer circumference. In the present manuscript, this expression is introduced as a literature‐derived mechanistic framework for interpreting suction‐associated interfacial fixation under wet contact conditions, rather than as a direct fitting model for the current clinical dataset. Although the formulation includes both suction‐related and capillary‐related terms, the suction‐associated component is particularly relevant to the present octopus‐inspired microcavity design because preload‐dependent deformation and subsequent vacuum establishment are central to its intended attachment mechanism [20].

Within this framework, the observed increases in adhesion‐related performance, penetration depth, hydration, and clinical skin appearance are consistent with the possibility that suction‐associated microstructural interaction contributed to the overall patch‐assisted response. At the same time, because a flat non‐suction occlusive patch was not included as a separate control, the present study does not directly resolve the relative contributions of micro‐suction, passive occlusion, and prolonged interfacial retention. Therefore, the current data support an a combined cupping stimulation and hydration retention effect under wet application conditions, while the independent mechanistic contribution of the suction architecture remains to be more rigorously isolated in future studies.

### 3.2 Adhesion Characteristics

To elucidate the suction‐mediated adhesion mechanism of the microstructured suction‐type patch under physiologically relevant conditions, we first directly evaluated the negative pressure generated by the microcavities upon contact, followed by an assessment of the resulting normal adhesion strength using cadaveric human skin as the testing substrate.

As shown in Figure 2a, the patch generated a measurable negative pressure immediately upon contact under the ampule‐treated (CNP) condition, which was stably maintained throughout the entire 15‐min application period. In contrast, the dry control condition exhibited negligible negative pressure over the same duration. Importantly, all measurements were conducted without any external vacuum source or active pressure control, confirming that the sustained suction originates solely from the passive microcavity architecture of the patch. Detailed information regarding the experimental setup and pressure measurement configuration is provided in the Figure S1.

Following this direct validation of sustained negative pressure generation, the normal adhesion strength of the patch was quantitatively evaluated on cadaveric human skin, rather than on simplified skin‐mimicking substrates. The use of cadaver skin enables closer representation of the native multilayered structure, surface topography, and viscoelastic properties of human skin. Normal adhesion measurements were performed using a custom‐designed universal testing machine equipped with 3 mm diameter suction probes.

As shown in Figure 2b, the patch exhibited significantly stronger normal adhesion under the ampule‐treated (CNP) condition compared to the dry control condition. The enhanced adhesion under moist conditions indicates that surface hydration synergistically interacts with suction‐induced negative pressure, promoting improved conformal contact and interfacial fixation on real skin tissue.

Collectively, these results demonstrate that the biomimetic microcavities, inspired by octopus suckers, generate localized and sustained suction that enables robust adhesion on human skin under physiologically relevant conditions. Beyond mechanical fixation, sustained passive negative pressure and cupping‐driven structural deformation have been reported to induce lateral separation between corneocyte layers and nanoscale changes in the stratum corneum, thereby facilitating increased permeability through the stratum corneum [20]. Such a suction‐induced microenvironment supports passive diffusion pathways and improved agent retention, forming a mechanistic basis for the suction‐type patch's functional and clinical performance. The downstream implications of this mechanism are further evaluated in the following sections using active ampule‐loaded formulations.

### 3.3 Penetration Depth

Figure 3 presents a depth‐resolved comparison of intradermal penetration with and without the suction‐type patch based on in vivo confocal z‐stack analysis. In the absence of the patch (Without Patch), the fluorescent tracer was primarily confined to the upper epidermal region, resulting in a relatively shallow penetration depth. In contrast, application of the suction‐type patch (With Patch) led to an expanded spatial distribution of the tracer into deeper skin layers, accompanied by a statistically significant increase in the mean penetration depth (Figure 3a). This difference was consistently observed in both the three‐dimensional confocal reconstructions and the corresponding cross‐sectional (XZ) views (Figure 3b,c).

These findings provide spatially resolved and quantitative evidence of altered intradermal distribution, rather than relying solely on indirect clinical outcomes. Confocal penetration depth analysis has been widely adopted in transdermal delivery research as a meaningful indicator to evaluate changes in delivery efficiency by directly visualizing the extent of material transport within skin tissue.

In the broader transdermal delivery literature, suction‐, cupping‐, or mechanically assisted systems have been reported to enhance skin penetration through transient modulation of the skin barrier, particularly the stratum corneum. Some studies have described this phenomenon using diffusion‐based quantitative models, showing that mechanically induced pressure or deformation can increase effective transport pathways or diffusion‐related parameters. Other studies have employed confocal imaging, histological analysis, or morphological observations to interpret increased penetration depth itself as direct spatial evidence of altered delivery behavior. In particular, prior octopus‐inspired architecture studies using hole or flat control structures have shown that protuberance‐containing suction structures enhance negative‐pressure generation, adhesion, and delivery‐related performance compared with less structured or flat interfaces [22]. Within this framework, Suction‐associated fixation and occlusive sealing are interpreted as coupled interfacial functions of the octopus‐inspired microcavity architecture. These coupled functions promote formulation retention and cupping stimulation, thereby supporting the enhanced intradermal distribution observed in patch‐assisted application. Collectively, these studies support the view that mechanically assisted patch interfaces can alter intradermal distribution under defined study conditions, although the individual magnitude of each contributing mechanism may differ across systems and experimental designs [20, 24, 25, 31, 32, 33, 34, 35].

In this context, the increased penetration depth observed in Figure 3 is interpreted as a result of coupled patch‐assisted interfacial functions under the tested moist application conditions. The confocal results indicate that the patch–ampule system produced a measurable change in intradermal tracer distribution compared with ampule application alone. Within this system, occlusive sealing and suction‐associated mechanics jointly supported enhanced formulation contact and intradermal distribution. Because a flat non‐suction occlusive patch was not included as a separate control, the individual magnitude of each contribution could not be separately quantified in the present design. Therefore, the confocal analysis presented here should be interpreted as direct spatial evidence of enhanced intradermal distribution within the tested patch–ampule system, rather than as isolated proof that suction alone accounts for the observed transport enhancement.

### 3.4 Anti‐Wrinkle Effect (Eye Area, 2 Weeks)

As shown in Figure 4, 3D surface imaging of the eye area was conducted using Antera 3D to assess wrinkle depth before and after 2 weeks of product application. Panels a and c represent the control group prior to and following treatment with the ampule alone, while panels b and d correspond to the treatment group that received both ampule and suction‐type patch application.

In the control group (a, c), the wrinkle depth appears slightly changed, with persistent warm color regions indicating consistent presence of deeper wrinkles. In contrast, the treatment group (b, d) exhibits a noticeable reduction in warm‐toned regions after 2 weeks, particularly around the nasojugal fold, suggesting shallower wrinkle contours. This visual mapping highlights the potential synergy between the suction‐type patch and the topical ampule in alleviating wrinkle depth under the tested patch–ampule application conditions.

The topographical changes in color distribution—transitioning from red‐orange to yellow‐green—indicate localized improvements in skin surface smoothness. These findings provide visual evidence of anti‐wrinkle efficacy and support the interpretation that the patch‐assisted condition was associated with wrinkle improvement within the tested patch–ampule system over the short‐term treatment window.

To complement the qualitative wrinkle mapping, Figure 5 presents quantitative analyses of wrinkle depth changes over the 2‐week treatment period. In panel **a**, the line graph illustrates the average wrinkle depth (mm) in the eye area for both control and treatment groups before and after the intervention. While both groups show slight decreases, the reduction in the treatment group is more pronounced. Panel b shows the Δ (delta) change in wrinkle depth as a bar graph. The treatment group demonstrates a statistically significant reduction compared to the control group, with a larger absolute change in average wrinkle depth (*p* < 0.01). This numerical evaluation corroborates the visual findings from Figure 4 and confirms the anti‐wrinkle efficacy of the suction‐type patch when used in conjunction with the ampule. Collectively, the data from Figure 5 support the hypothesis that patch‐assisted application not only enhances adhesion and skin contact but also promotes measurable clinical improvement in wrinkle parameters within a short‐term window.

As shown in Table 1, the mean wrinkle depth in the test group decreased from 0.1230 ± 0.0214 mm at baseline to 0.1098 ± 0.0197 mm after 2 weeks, representing a 10.74% improvement. In contrast, the control group exhibited a smaller reduction from 0.1068 ± 0.0213 mm to 0.1001 ± 0.0211 mm, corresponding to a 6.27% improvement. Statistical analysis revealed significant intragroup changes in both groups (*p* = 0.005), while the intergroup comparison using Generalized Estimating Equations (GEE) confirmed a significant difference favoring the test group (*p* < 0.001). The calculated relative improvement in wrinkle depth between the groups reached 71.40%, highlighting the enhanced efficacy of the suction‐type microstructured patch in conjunction with topical ampule application. These findings from Table 1 provide quantitative validation of the anti‐wrinkle benefit observed visually in Figures 4 and 5.

The wrinkle‐reducing effect observed in this study may reflect an integrated patch‐assisted response involving conformal contact, hydration support, prolonged residence of the applied formulation, and possible suction‐associated interfacial interaction under moist application conditions. The suction‐type microstructured patch physically adheres to the skin with uniform and stable contact, forming localized negative pressure that may contribute to reversible nanoscale structural modulation at the stratum corneum interface [20]. These factors may support improved local retention of the applied formulation and hydration‐related improvement within the tested patch–ampule system, both of which may contribute to reduced wrinkle depth by improving the superficial skin condition. Suction‐associated fixation and occlusive sealing are therefore interpreted as the principal coupled functions of the octopus‐inspired microcavity architecture within the tested patch–ampule system. These coupled functions produce hydration support and cupping stimulation, thereby contributing to the integrated patch‐assisted response observed in the present clinical design.

Additionally, the patch's microcavity‐induced negative pressure may have redistributed local tension on the skin, smoothing out fine lines and improving skin texture. Combined with the cosmeceutical ampule applied prior to patch placement, this setup likely enhanced the delivery and retention of moisturizing and bioactive compounds, further supporting dermal matrix hydration and elasticity. These results are consistent with prior observations that occlusive systems can transiently reduce wrinkle appearance by improving hydration and soft tissue pliability. However, the observed 71.40% relative improvement in wrinkle depth in the test group suggests that the patch‐assisted condition provided an enhanced anti‐wrinkle response within the tested patch–ampule system. Suction‐associated fixation and occlusive sealing are therefore interpreted as coupled functions of the octopus‐inspired microcavity architecture within the tested patch–ampule system. These coupled functions produce hydration support and cupping stimulation, thereby jointly supporting the observed anti‐wrinkle response. Therefore, the combination of mechanical sealing and moisture synergy underlies the clinically meaningful wrinkle improvement demonstrated in this 2‐week trial, as visualized in Figure 4 and quantified in Table 1. The paired between‐condition difference in change was 0.00652 (95% CI, 0.00404–0.00900; Cohen's dz. = 1.88), indicating a large observed effect favoring the patch‐assisted condition.

### 3.5 Skin Elasticity Improvement

Figure 6 presents the results of skin elasticity measurements based on CoR (Coefficient of Restitution) values. In Figure 6a, a line graph illustrates the change in elasticity before and after 2 weeks of application, comparing the test group using both the suction‐type patch and ampule with the control group using ampule alone. While both groups showed a slight upward trend, the test group exhibited a more notable improvement in skin elasticity.

Figure 6b quantitatively depicts the ΔCoR (change in elasticity) as a bar graph, where the test group showed a significantly greater increase in CoR value compared to the control. These results indicate that the suction‐type patch contributed to enhanced skin elasticity, likely due to the combined effects of skin tension modulation and improved moisture retention from enhanced adhesion and occlusion. The statistical significance (**p < 0.05) supports the efficacy of the patch‐assisted application in restoring skin resilience.

As shown in Table 2, skin elasticity was quantitatively assessed using the Coefficient of Restitution (CoR), a parameter reflecting the skin's ability to rebound after deformation. Both groups started with identical baseline values (0.5900), confirming comparable initial conditions. After 2 weeks of product use, the test group exhibited an increase in elasticity to 0.6200 ± 0.0400, while the control group showed a smaller rise to 0.6000 ± 0.0500.

**TABLE 2:** Skin elasticity (CoR) values.

	Test group cheek, skin elasticity (CoR)	Control group cheek, skin elasticity (CoR)
Baseline	0.5900 ± 0.0400	0.5900 ± 0.0500
After 2 weeks	0.6200 ± 0.0400	0.6000 ± 0.0500
Intragroup significance^a^ (baseline vs. after 2 weeks)	0.005	0.006
Intragroup significance^b^	0.001	
Improvement (%) (baseline vs. after 2 weeks)	4.11%	2.03%
Relative improvement	102.66%	

^a^ *p* value: significant probability, Wilcoxon signed rank test (*p* < 0.05, comparison to initial value).

^b^ *p* value: significant probability, Generalized Estimating Equations (*p* < 0.05, comparison between groups).

Statistical analysis indicated significant intragroup improvements for both the test (*p* = 0.005) and control (*p* = 0.006) groups. However, intergroup comparison using Generalized Estimating Equations (GEE) revealed a statistically significant difference favoring the test group (*p* = 0.001). The percentage improvement was 4.11% for the test group versus 2.03% for the control group, corresponding to a 102.66% relative enhancement. These findings confirm that the suction‐type patch contributed meaningfully to improved skin elasticity compared to ampule use alone. The paired between‐condition difference in change was 0.0110 (95% CI, 0.00572–0.01628; Cohen's dz. = 1.49), again indicating a large observed effect in favor of the patch‐assisted condition.

The improvement in skin elasticity observed in the test group is interpreted as an integrated patch‐assisted response driven by the coupled functions of Suction‐associated fixation and occlusive sealing provided by the octopus‐inspired microcavity architecture under the tested moist application conditions. These coupled functions promote conformal contact, hydration retention, prolonged formulation residence, local interfacial stability, and cupping stimulation within the tested patch–ampule system. In the present study, the increased CoR values are most appropriately interpreted as evidence of elasticity‐related improvement within this coupled suction–occlusion interface. Because a flat non‐suction occlusive patch was not included as a separate control, the individual magnitude of each contribution could not be separately quantified in the current design.

### 3.6 Skin Moisturization Enhancement

Figure 7 illustrates representative hydration maps obtained using the Epsilon E100 device, highlighting changes in skin moisture retention on the cheek area. Images (a) and (b) show baseline hydration levels of the control and treatment groups, respectively, prior to product application. After 2 weeks of use, images (c) and (d) demonstrate the posttreatment hydration status for the control and treatment groups. The treatment group (d), which applied both the ampule and the suction‐type patch, exhibits noticeably brighter and denser blue coloration compared to its baseline (b) and to the control group (c), indicating a significant enhancement in skin moisture content. The visual data in Figure 7 support the interpretation that the patch‐assisted condition was associated with greater hydration retention under the tested study conditions.

Figure 8 presents a quantitative assessment of skin hydration using the dielectric constant (ε) measured by the Epsilon E100 device. In panel (a), the test group shows a marked increase in average ε value after 2 weeks of product application, while the control group displays only a modest rise. This suggests that the combination of the ampule and suction‐type patch led to enhanced moisture retention at the skin surface. Panel (b) further quantifies the improvement, showing the Δε (difference between pre and posttreatment values) for each group. The treatment group exhibits a significantly greater Δε compared to the control, confirming greater hydration improvement in the patch‐assisted condition under the tested study conditions.

These findings from Table 3 quantitatively support that the patch‐assisted condition produced greater hydration improvement than ampule application alone under the tested study conditions. However, this increased hydration should be interpreted cautiously. The greater increase in hydration observed in the patch‐assisted condition may reflect a combined effect of conformal occlusion, moisture retention, prolonged residence of the applied formulation, and suction‐associated microstructural interaction under moist contact conditions. The hydration improvement is therefore interpreted as being driven by the coupled functions of Suction‐associated fixation and occlusive sealing provided by the octopus‐inspired microcavity architecture. These coupled functions produce moisture retention and cupping stimulation, thereby supporting enhanced skin moisturization within the tested patch–ampule system, although their individual magnitudes were not separately quantified in the present clinical design.

**TABLE 3:** The result by average dielectric constant.

	Test group cheek, average dielectric constant (ε)	Control group cheek, average dielectric constant (ε)
Baseline	36.80 ± 7.30	36.10 ± 7.31
After 2 weeks	47.34 ± 6.44	40.16 ± 7.44
Intragroup significance^a^ (baseline vs. after 2 weeks)	0.005	0.005
Intragroup significance^b^	< 0.001	
Improvement (%) (baseline vs. after 2 weeks)	28.63%	11.24%
Relative improvement	154.71%	

^a^ *p* value: significant probability, Wilcoxon signed rank test (*p* < 0.05, comparison to initial value).

^b^ *p* value: significant probability, Generalized Estimating Equations (*p* < 0.05, comparison between groups).

The pronounced improvement in skin moisturization observed in the test group is therefore interpreted as being driven by the coupled functions of Suction‐associated fixation and occlusive sealing provided by the octopus‐inspired microcavity architecture. These coupled functions reduce transepidermal water loss and maintain close contact between the formulation and the skin surface, thereby producing moisture retention, hydration support, and cupping stimulation within the tested patch–ampule system. Thus, the present hydration result should be understood as evidence of improved moisturization within this coupled suction–occlusion interface, although the individual magnitude of each contribution could not be separately quantified in the current study design.

Such hydration enhancement is interpreted as being driven by the coupled functions of Suction‐associated fixation and occlusive sealing provided by the octopus‐inspired microcavity architecture under moist contact conditions. These coupled functions promote moisture retention, prolonged formulation residence, hydration support, and cupping stimulation within the tested patch–ampule system. In addition, the concurrent application of a bioactive ampule supplied moisturizing ingredients, further supporting local hydration. Thus, the hydration result should be interpreted as an integrated patch‐assisted response driven by the joint action of Suction‐associated fixation and occlusive sealing, although the individual magnitude of each contribution was not separately quantified in the present clinical design. The paired between‐condition difference in change was 6.479 (95% CI, 4.893–8.065; Cohen's dz. = 2.92), indicating a very large observed effect for the patch‐assisted condition.

### 3.7 Reduction in Pigmentation (Melanin Concentration, 4 Weeks)

Figure 9 illustrates melanin distribution changes in the cheek area over a 4‐week period using DermaVision imaging. Images a and c show the control group before and after treatment, respectively, while images b and d correspond to the test group, which received both the suction‐type patch and ampule. In image a, the control group initially presents with moderate melanin intensity, and in image c, only slight visual improvement is observed. In contrast, image b of the treatment group shows intense pigmentation at baseline, which is markedly reduced after 4 weeks as seen in image d. This visual reduction in hyperpigmented regions suggests that the patch‐assisted condition was associated with greater melanin reduction within the tested patch–ampule system compared to ampule application alone. The results in Figure 9 align with the quantitative pigmentation reduction trends observed in the subsequent data.

Figure 10 presents quantitative changes in skin pigmentation over the 4‐week treatment period. In panel a, the line graph depicts the mean melanin color values before and after application for both the test and control groups. While both groups show a decrease, the reduction is visibly more pronounced in the test group that used the suction‐type patch and ampule together. Panel b shows the Δ (delta) melanin color percentage change between groups, clearly indicating a significantly greater reduction in pigmentation in the test group compared to the control. These results, supported by statistical significance (**p < 0.05), validate the effectiveness of the patch‐assisted application in promoting pigment lightening. Figure 10 therefore reinforces the observation that the patch‐assisted condition was associated with greater pigmentation improvement under the tested study conditions.

As shown in Table 4, the melanin color value in the test group decreased from 80.21% ± 13.67% at baseline to 67.56% ± 18.63% after 4 weeks of application, resulting in a 15.77% reduction. In contrast, the control group exhibited a smaller decrease from 85.78% ± 11.91% to 81.50% ± 14.57%, corresponding to only a 4.99% improvement. Statistical tests confirmed significant intragroup changes in both groups (*p* = 0.012 for test, *p* = 0.028 for control), while the intergroup difference was also significant (*p* < 0.012). These results indicate that the combination of suction‐type patch and ampule yielded more pronounced pigmentation reduction than ampule alone. The calculated relative improvement reached 216.09%, strongly supporting the enhanced performance of the patch‐assisted delivery system in skin tone correction.

**TABLE 4:** Pigmentation improvement table.

	Test group cheek, melanin color (%)	Control group cheek, melanin color (%)
Baseline	80.21 ± 13.67	85.78 ± 11.91
After 4 weeks	67.56 ± 18.63	81.50 ± 14.57
Intragroup significance^a^ (baseline vs. after 4 weeks)	0.012	0.028
Intragroup significance^b^	0.012	
Improvement (%) (baseline vs. after 4 weeks)	15.77%	4.99%
Relative improvement	216.09%	

^a^ *p* value: significant probability, Wilcoxon signed rank test (*p* < 0.05, comparison to initial value).

^b^ *p* value: significant probability, Generalized Estimating Equations (*p* < 0.05, comparison between groups).

The observed reduction in pigmentation in the treatment group should be interpreted with appropriate caution. Rather than direct proof of isolated suction‐driven delivery enhancement, the pigmentation result is interpreted as an integrated patch‐assisted response driven by the coupled functions of Suction‐associated fixation and occlusive sealing under moist application conditions. These coupled functions promote improved local hydration, prolonged retention of the applied formulation, more sustained and uniform exposure of the skin to the formulation, and cupping stimulation within the tested patch–ampule system. Thus, the present pigmentation result is most appropriately interpreted as formulation‐specific evidence of improved skin tone modulation driven by the joint action of Suction‐associated fixation and occlusive sealing, although the individual magnitude of each contribution was not separately quantified in the present clinical design. The paired between‐condition difference in change was 8.37 (95% CI, 3.43–13.31; Cohen's dz. = 1.21), indicating a large observed effect favoring the patch‐assisted condition.

## 4 Conclusion

In this study, we conducted a pilot‐scale clinical evaluation of a pre‐developed biomimetic suction‐type microstructured patch used in combination with a functional cosmeceutical ampule. Over a 4‐week split‐face human trial involving 10 participants, the patch‐assisted application was associated with multidimensional improvements in skin parameters, including wrinkle depth, elasticity, hydration, and pigmentation. These results demonstrate that clinically measurable improvement effects, as reflected by quantitative outcome metrics such as wrinkle reduction and hydration enhancement, are indeed present upon patch‐assisted application. These effects are attributed in part to the patch's octopus‐inspired microcavity design, which enhanced skin adhesion, promoted occlusive conditions, and supported localized retention of active ingredients.

Quantitative imaging and instrumental measurements revealed statistically significant differences favoring the patch‐treated side compared to ampule application alone, without any observed adverse reactions. These findings suggest that suction‐based architectural interfaces may facilitate both mechanical skin modulation and improved topical treatment efficacy under noninvasive and skin‐conformable conditions. However, given the small sample size, short evaluation period, and limited population diversity, the generalizability of these results remains constrained. Because the present study evaluated a specific commercial ampule formulation in combination with the suction‐type patch, the observed clinical responses should be interpreted as formulation‐specific pilot findings. Differences in composition, active ingredients, solvent system, viscosity, and interfacial behavior across other topical formulations may influence the extent to which similar patch‐assisted effects are observed. Accordingly, broader generalization to other cosmetic or dermatological formulations should be made with caution.

It should be noted that various physical enhancement technologies for transdermal delivery, including microneedle patches, suction‐based systems, hypobaric devices, and other mechanically or energy‐assisted approaches, have been reported in the literature [24, 25]. Previous studies have already demonstrated quantitative improvements in transdermal transport or clinical skin parameters using these diverse technologies under their respective experimental conditions. However, these strategies differ fundamentally in terms of invasiveness, application duration, dosing regimens, delivery mechanisms, and skin recovery profiles. As a result, although favorable performance trends relative to prior reports can be identified in certain aspects, direct numerical benchmarking across studies conducted under different conditions remains inherently limited and may not be scientifically appropriate [24, 36].

In this context, the suction‐type biomimetic patch presented here represents a distinct, noninvasive delivery‐assisting strategy that operates through short‐duration application and passive, structure‐mediated mechanical interaction without the need for external energy input. Rather than aiming to outperform existing technologies under identical numerical endpoints, the present results should be interpreted as evidence that bioinspired suction‐based interfaces can meaningfully enhance topical treatment efficacy within a consumer‐friendly and skin‐conformable usage framework.

Overall, the present pilot study supports the feasibility of a suction‐type microstructured patch as a noninvasive, short‐duration, patch‐assisted cosmetic application platform associated with measurable improvements in wrinkles, elasticity, hydration, and pigmentation. However, these findings should be interpreted with appropriate caution. Because a flat non‐suction occlusive patch was not included as separate control, the present design could not fully isolate the independent contribution of the suction microstructure from nonsuction occlusion, hydration retention, or prolonged interfacial residence effects. Therefore, the current results are best interpreted as evidence of a combined cupping stimulation and hydration retention effect under moist application conditions, while the relative mechanistic contribution of suction itself remains to be clarified in future studies directly comparing the suction‐type microstructured patch with a flat non‐suction occlusive patch of comparable material and geometry.

## Author Contributions

S.C. and H.‐P. contributed equally to this work. S.C. was responsible for conceptualizing the patch structure, developing the fabrication methodology, and drafting the initial manuscript. H.‐P. performed the majority of the in vitro and in vivo experiments and contributed significantly to data acquisition and analysis. Both authors were equally involved in critical discussions throughout the study and share first authorship. J.A. supported clinical measurements and contributed to data interpretation. D.‐H.K., J.‐H.K., and B.W.H. assisted in developing the clinical protocols and coordinating the human study. S.N. and W.H. contributed to the clinical study design and to the interpretation of the clinical results. K.H.L. supervised the industrial implementation of the study, coordinated experimental validation, and critically revised the manuscript. D.W.K. conceived and academically led the project, interpreted the scientific findings, structured the manuscript, and completed the final revisions. K.H.L. and D.W.K. share corresponding authorship and were equally responsible for overseeing the research and manuscript development. All authors have read and approved the final version of the manuscript.

## Funding

The authors gratefully acknowledge the support from the industry‐academia cooperation research of Mimetics Co. Ltd. Additional funding was provided by Program through the Korea Institutes of Police Technology (KIPoT) funded by the Korean National Police Agency & Korea Customs Service (RS‐2024‐00406234). This work was partly supported by the Technology development Program of MSS S3407896 and the ICT development R&D program of MSIT S3407896.

## Ethics Statement

The authors confirm that the ethical policies of the journal, as noted on the journal's author guidelines page, have been adhered to. All subjects voluntarily participated and signed a written informed consent. Additionally, informed consent was obtained from all participants for the use of their photographs.

## Consent

Informed consent was obtained from all subjects involved in the study.

## Conflicts of Interest

The authors declare no conflicts of interest.

## Supporting information


**Figure S1:** Detailed experimental setup for direct quantification of negative pressure generated by the microstructured suction‐type patch.
**Figure S2:** Representative Antera 3D modality images: (a) skin color, (b) skin texture, (c) wrinkle depth, (d) pore density, (e) melanin concentration, and (f) hemoglobin distribution. These six imaging modes allow for multiparametric visualization of skin conditions before and after patch application.
**Figure S3:** Representative Epsilon E100 capacitance‐based hydration imaging: (a) skin color map, (b) skin texture map, and (c) wrinkle‐associated dielectric map. Brighter areas correspond to higher dielectric constant (ε) values, indicating increased skin moisture content.
**Figure S4:** DermaVision multimodal pigmentation analysis: (a) cross‐polarized imaging (CPI) for superficial pigmentation, (b) parallel‐polarized imaging (PPI) for texture‐related melanin distribution, and (c) UV imaging for deep epidermal pigmentation assessment.

## Data Availability

The data that support the findings of this study are available on request from the corresponding author. The data are not publicly available due to privacy or ethical restrictions.

## References

[1] Z. D. Draelos , “The Cosmeceutical Realm,” Clinics in Dermatology 26, no. 6 (2008): 627–632. 18940543 10.1016/j.clindermatol.2007.09.005

[2] W. Y. Jeong , M. Kwon , H. E. Choi , and K. S. Kim , “Recent Advances in Transdermal Drug Delivery Systems: A Review,” Biomaterials Research 25, no. 1 (2021): 24. 34321111 10.1186/s40824-021-00226-6 PMC8317283

[3] S. M. Mortazavi and H. R. Moghimi , “Skin Permeability, a Dismissed Necessity for Anti‐Wrinkle Peptide Performance,” International Journal of Cosmetic Science 44, no. 2 (2022): 232–248. 35302659 10.1111/ics.12770

[4] J. A. Bouwstra and M. Ponec , “The Skin Barrier in Healthy and Diseased State,” Biochimica et Biophysica Acta, Biomembranes 1758, no. 12 (2006): 2080–2095. 10.1016/j.bbamem.2006.06.021 16945325

[5] M. Avcil , G. Akman , J. Klokkers , D. Jeong , and A. Çelik , “Efficacy of Bioactive Peptides Loaded on Hyaluronic Acid Microneedle Patches: A Monocentric Clinical Study,” Journal of Cosmetic Dermatology 19, no. 2 (2020): 328–337. 31134751 10.1111/jocd.13009

[6] K. K. Hendel , C. Bagger , U. H. Olesen , et al., “Fractional Laser‐Assisted Topical Delivery of Bleomycin Quantified by LC‐MS and Visualized by MALDI Mass Spectrometry Imaging,” Drug Delivery 26, no. 1 (2019): 244–251. 30859849 10.1080/10717544.2019.1574937 PMC6419659

[7] K. Ita , “Transdermal Delivery of Drugs With Microneedles—Potential and Challenges,” Pharmaceutics 7, no. 3 (2015): 90–105. 26131647 10.3390/pharmaceutics7030090 PMC4588187

[8] H. Lv , N. Gao , Q. Zhou , Y. Wang , G. Ling , and P. Zhang , “Collagen‐Based Dissolving Microneedles With Flexible Pedestals: A Transdermal Delivery System for Both Anti‐Aging and Skin Diseases,” Advanced Healthcare Materials 12, no. 21 (2023): 2203295. 10.1002/adhm.202203295 37029522

[9] P. Makvandi , M. Kirkby , A. R. J. Hutton , et al., “Engineering Microneedle Patches for Improved Penetration: Analysis, Skin Models and Factors Affecting Needle Insertion,” Nano Micro Letters 13, no. 1 (2021): 93. 34138349 10.1007/s40820-021-00611-9 PMC8006208

[10] Z. Sartawi , C. Blackshields , and W. Faisal , “Dissolving Microneedles: Applications and Growing Therapeutic Potential,” Journal of Controlled Release 348 (2022): 186–205. 35662577 10.1016/j.jconrel.2022.05.045

[11] J. Y. Shin , D. Han , K. Y. Yoon , D. H. Jeong , and Y. I. Park , “Clinical Safety and Efficacy Evaluation of a Dissolving Microneedle Patch Having Dual Anti‐Wrinkle Effects With Safe and Long‐Term Activities,” Annals of Dermatology 36, no. 4 (2024): 215–224. 39082657 10.5021/ad.23.136 PMC11291098

[12] P. Te , J. Meephansan , P. Sirithanabadeekul , et al., “A Split‐Face Comparison of Novel Microneedle Patch Versus Botulinum Toxin‐A and Microneedle Patch for Improvement in Undereye Skin Texture,” Cosmetics 11, no. 3 (2024): 100.

[13] S. Baik , D. W. Kim , Y. Park , T.‐J. Lee , S. Ho Bhang , and C. Pang , “A Wet‐Tolerant Adhesive Patch Inspired by Protuberances in Suction Cups of Octopi,” Nature 546, no. 7658 (2017): 396–400. 28617467 10.1038/nature22382

[14] S. Baik , J. Kim , H. J. Lee , T. H. Lee , and C. Pang , “Highly Adaptable and Biocompatible Octopus‐Like Adhesive Patches With Meniscus‐Controlled Unfoldable 3D Microtips for Underwater Surface and Hairy Skin,” Advanced Science 5, no. 8 (2018): 1800100. 30128235 10.1002/advs.201800100 PMC6097001

[15] Y.‐C. Chen and H. Yang , “Octopus‐Inspired Assembly of Nanosucker Arrays for Dry/Wet Adhesion,” ACS Nano 11, no. 6 (2017): 5332–5338. 28448714 10.1021/acsnano.7b00809

[16] Y. Guo , X. Wang , L. Zhang , et al., “From Dry to Wet, the Nature Inspired Strong Attachment Surfaces and Their Medical Applications,” ACS Nano 19, no. 10 (2025): 9684–9708. 40051147 10.1021/acsnano.4c17864 PMC11924587

[17] J.‐H. Hsu , N.‐T. Tang , T.‐F. Hsu , et al., “Self‐Assembly of *Hemimyzon formosanus* ‐Inspired Crescent‐Shaped Nanosucker Arrays for Reversible Adhesion,” ACS Applied Materials & Interfaces 15, no. 48 (2023): 56203–56212. 38009758 10.1021/acsami.3c15577

[18] D. Lim , M. W. Jeong , H. Min , et al., “Autonomous Self‐Healing 3D Micro‐Suction Adhesives for Multi‐Layered Amphibious Soft Skin Electronics,” InfoMat 6, no. 10 (2024): e12603.

[19] M. Bok , Z.‐J. Zhao , S. H. Hwang , et al., “Biocompatible All‐In‐One Adhesive Needle‐Free Cup Patch for Enhancing Transdermal Drug Delivery,” ACS Applied Materials & Interfaces 13, no. 48 (2021): 58220–58228. 34793117 10.1021/acsami.1c18750

[20] J. Lee , G. W. Hwang , B. S. Lee , et al., “Artificial Octopus‐Limb‐Like Adhesive Patches for Cupping‐Driven Transdermal Delivery With Nanoscale Control of Stratum Corneum,” ACS Nano 18, no. 7 (2024): 5311–5321. 10.1021/acsnano.3c09304 38254288

[21] R. Huang , X. Zhang , W. Li , L. Shang , H. Wang , and Y. Zhao , “Suction Cups‐Inspired Adhesive Patch With Tailorable Patterns for Versatile Wound Healing,” Advanced Science 8, no. 17 (2021): 2100201. 34196481 10.1002/advs.202100201 PMC8425934

[22] M. Song , H.‐K. Park , M. Kim , et al., “Skin‐Adaptive Nanofiber‐Based Adhesive Electronics With Octopus‐Like 3D Suction Cups for Enhanced Transdermal Delivery,” npj Flexible Electronics 9, no. 1 (2025): 54.

[23] G. R. Kang , G. W. Hwang , D. Lim , et al., “Robustly Repeatable, Permeable, and Multi‐Axially Stretchable, Adhesive Bioelectronics With Super‐Adaptive Conductive Suction Cups for Continuously Deformable Biosurfaces,” Advanced Science 12, no. 25 (2025): 2500346. 40162820 10.1002/advs.202500346 PMC12224995

[24] D. Ramadon , M. T. C. McCrudden , A. J. Courtenay , and R. F. Donnelly , “Enhancement Strategies for Transdermal Drug Delivery Systems: Current Trends and Applications,” Drug Delivery and Translational Research 12 (2022): 758–791. 33474709 10.1007/s13346-021-00909-6 PMC7817074

[25] F. Benaouda , R. Inacio , C. H. Lim , et al., “Needleless Administration of Advanced Therapies Into the Skin via the Appendages Using a Hypobaric Patch,” Proceedings of the National Academy of Sciences of the United States of America 119, no. 18 (2022): e2120340119. 35482922 10.1073/pnas.2120340119 PMC9170139

[26] A. A. Alsharif , A. M. Syed , X. Li , N. A. Alsharif , G. Lubineau , and N. El‐Atab , “Hybrid 3D Printing of a Nature‐Inspired Flexible Self‐Adhesive Biopatch for Multi‐Biosignal Sensing,” Advanced Functional Materials 34, no. 44 (2024): 2406341.

[27] Z. Zhu , J. Wang , X. Pei , et al., “Blue‐Ringed Octopus‐Inspired Microneedle Patch for Robust Tissue Surface Adhesion and Active Injection Drug Delivery,” Science Advances 9, no. 25 (2023): eadh2213. 37343097 10.1126/sciadv.adh2213 PMC10284554

[28] Z. Luo , D. Klein Cerrejon , S. Römer , N. Zoratto , and J.‐C. Leroux , “Boosting Systemic Absorption of Peptides With a Bioinspired Buccal‐Stretching Patch,” Science Translational Medicine 15, no. 715 (2023): eabq1887. 37756378 10.1126/scitranslmed.abq1887

[29] M. Zare , E. R. Ghomi , P. D. Venkatraman , and S. Ramakrishna , “Silicone‐Based Biomaterials for Biomedical Applications: Antimicrobial Strategies and 3D Printing Technologies,” Journal of Applied Polymer Science 138, no. 38 (2021): 50969.

[30] M. Singh , D. L. Teodorescu , M. Rowlett , et al., “A Tunable Soft Silicone Bioadhesive for Secure Anchoring of Diverse Medical Devices to Wet Biological Tissue,” Advanced Materials 36, no. 3 (2024): 2307288. 10.1002/adma.202307288 PMC11801177 37865838

[31] S. Li , Q. Chen , Y. Zhang , et al., “Hyaluronic Acid Dissolving Microneedle Patch‐Assisted Acupoint Transdermal Delivery of Triptolide for Effective Rheumatoid Arthritis Treatment,” Scientific Reports 14 (2024): 25256. 39448702 10.1038/s41598-024-76341-w PMC11502756

[32] K. Al‐Japairai , S. H. Almurisi , F. Alheibshy , N. Abdul‐Halim , and S. Mahmood , “Rapid Dissolving Microneedle Patch Integrated With Benidipine‐Loaded Nanotransfersomes for Transdermal Drug Delivery: Optimization, Characterizations, and Preclinical Bioavailability Assessment,” Drug Delivery and Translational Research 16 (2025): 1629–1649. 41094229 10.1007/s13346-025-01976-9 PMC13038691

[33] Y. Oaku , T. Kuwae , T. Misono , T. Ogura , and A. Abe , “Evaluation of Skin Penetration of Fluorescent Dissolved Formulations Using Confocal Laser Scanning Microscopy,” Pharmaceutics 17 (2025): 1534. 41471050 10.3390/pharmaceutics17121534 PMC12736841

[34] Y. Zou , A. Celli , H. Zhu , et al., “Confocal Laser Scanning Microscopy to Estimate Nanoparticles' Human Skin Penetration In Vitro,” International Journal of Nanomedicine 12 (2017): 8035–8041. 29184403 10.2147/IJN.S139139 PMC5673047

[35] M. Kim , M. Song , J. Son , G. W. Hwang , H.‐K. Park , and C. Pang , “Effective HA‐Loading Strategy in Internal Dome‐Assisted Suction Cups for Enhanced Transdermal Delivery Under Vertical Adhesion and Vibration,” Advanced Functional Materials 36 (2025): e19672.

[36] S. Liu , T. Deng , H. Cheng , J. Lu , and J. Wu , “Advances in Transdermal Drug Delivery Systems and Clinical Applications in Inflammatory Skin Diseases,” Pharmaceutics 17 (2025): 746. 40574058 10.3390/pharmaceutics17060746 PMC12195946

