# Supplementary material for: A Pilot Clinical Study of a Biomimetic Suction Patch for Improving Wrinkles, Elasticity, Hydration, and Pigmentation via Enhanced Topical Delivery
Source: J Cosmet Dermatol. 2026 Jul 15;25(7):e71074. doi: 10.1111/jocd.71074 (PMC13373254; doi:10.1111/jocd.71074)
Supplement: Supplementary file 1 — Figure S1: Detailed experimental setup for direct quantification of negative pressure generated by the microstructured suction‐type patch. Figure S2: Representative Antera 3D modality images: (a) skin color, (b) skin texture, (c) wrinkle depth, (d) pore density, (e) melanin concentration, and (f) hemoglobin distribution. These six imaging modes allow for multiparametric visualization of skin conditions before and after patch application. Figure S3: Representative Epsilon E100 capacitance‐based hydration imaging: (a) skin color map, (b) skin texture map, and (c) wrinkle‐associated dielectric map. Brighter areas correspond to higher dielectric constant (ε) values, indicating increased skin moisture content. Figure S4: DermaVision multimodal pigmentation analysis: (a) cross‐polarized imaging (CPI) for superficial pigmentation, (b) parallel‐polarized imaging (PPI) for texture‐related melanin distribution, and (c) UV imaging for deep epidermal pigmentation assessment. [file JOCD-25-e71074-s001.docx]

**Supplementary Materials**

***A Pilot Clinical Study of a Biomimetic Suction Patch for Improving Wrinkles, Elasticity, Hydration, and Pigmentation via Enhanced Topical Delivery***

*Seunghoon Choi^1^, Hyung-ki Park^2^, Jaehwan Ahn^2^, Dong-Hyun Ko^3^, Jin-Hyun Kim^3^, Byung Woo Hwang^3^, Seongmin Noh^4^, Wonkyu Hong^5^, Keun Ho Lee^2^*, and Da Wan Kim^6^**

S. Choi
Department SKKU Advanced Institute of Nanotechnology (SAINT)

Sungkyunkwan University (SKKU)

2066 Seobu-ro, Jangan-gu, Suwon, Gyeonggi-do, 16419, Republic of Korea

H Park, J Ahn and K. H. Lee

Mimetics Co., Ltd, 2066 Seobu-ro, Jangan-gu, Suwon, 16419, Republic of Korea.

E-mail: khlee@mimetics.co.kr

D.-H. Ko, J.-H. Kim, B. W. Hwang

R&D Center, LG Household & Health Care (LG H&H), 70 Magokjungang 10-ro, Gangseo-gu, Seoul 07795, Republic of Korea.

S. Noh
Benjamin Clinic, 6F, 138 Dosan-daero, Gangnam-gu, Seoul 06040, Republic of Korea.

W. Hong
Human Clinical Skin Testing Center, 15F, Artis Forum, 62 Digital-ro 31-gil, Guro-gu, Seoul 08376, Republic of Korea

D. W. Kim

Department of Electronic Engineering

Korea National University of Transportation

Chungju-si, Chungbuk 27469, Republic of Korea

E-mail: dawankim@ut.ac.kr

**Keywords**: Biomimetic patch, Suction-based adhesion, Microstructured patch, Pilot clinical study, Wrinkle reduction, Skin elasticity, Skin hydration, Pigmentation, Cosmetic dermatology, Human evaluation

S. C, and H. P. contributed equally to this work.

Correspondce: K. H. Lee (khlee@mimetics.co.kr), and D. W. Kim (dawankim@ut.ac.kr)

**Supplementary Note**

***Ampule Formulation Information***

The cosmetic ampule used in this study was CNP Derma Answer Active Boost Ampule. The product was supplied by the study sponsor and used as the topical formulation in both the control and treatment conditions according to the same study protocol.

***Full ingredient composition***Water, Glycerin, Dipropylene Glycol, PEG/PPG/Polybutylene Glycol-8/5/3 Glycerin, Niacinamide, Bis-PEG-18 Methyl Ether Dimethyl Silane, Butylene Glycol, 1,2-Hexanediol, PEG-7 Glyceryl Cocoate, Dimethicone, Betaine, Jojoba Wax PEG-120 Esters, Methylpropanediol, Panthenol, PEG/PPG-17/6 Copolymer, Sodium DNA, Lactobacillus Ferment Lysate, Polymethylsilsesquioxane, Triethylhexanoin, Sodium Acrylate/Vinyl Alcohol Copolymer, Hydrogenated Lecithin, Sodium Polyacrylate, Lactobacillus/Soybean Ferment Extract, Chondrus Crispus Extract, Ceramide NP, Acrylates/C10-30 Alkyl Acrylate Crosspolymer, C14-22 Alcohols, Squalane, Tromethamine, Saccharomyces/Potato Extract Ferment Filtrate, Sodium Acrylic Acid/MA Copolymer, Saccharomyces/Barley Seed Ferment Filtrate, Sucrose Stearate, Rosmarinus Officinalis (Rosemary) Leaf Oil, C18-21 Alkane, Macadamia Ternifolia Seed Oil, Polyglyceryl-10 Stearate, Stearic Acid, Polyglyceryl-10 Oleate, Glyceryl Acrylate/Acrylic Acid Copolymer, Adenosine, Arachidyl Glucoside, Butyrospermum Parkii (Shea) Butter, Tocopherol, Palmitic Acid, Salvia Sclarea (Clary) Oil, Sodium Hyaluronate, Trisodium EDTA, Glycine Soja (Soybean) Oil, Trideceth-6, Cholesterol, Glyceryl Stearate, Troxerutin, Beta-Glucan, Carbomer, PEG-100 Stearate, Arginine, Glutamic Acid, Juniperus Mexicana Oil, Glycosphingolipids, Linalool, Limonene.

***Fabrication and Material Information of the Suction-Type Microstructured Patch***The suction-type microstructured patch was fabricated by Mimetics, Co., Ltd. (Suwon, Korea) using an injection molding process with liquid silicone. Briefly, liquid silicone (Dow Chemical Korea Limited) was mixed with a curing agent, both of which are biocompatible materials. The resulting mixture was injected into a molding system and thermally cured at temperatures above 100 °C for several seconds to produce the final patch structure [1, 2].

**
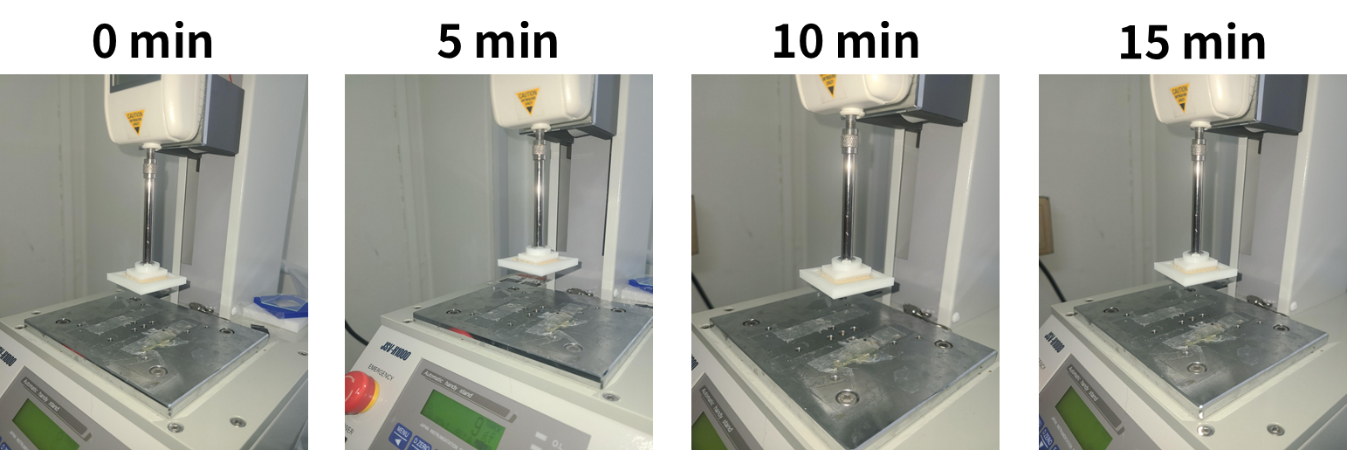
**

**Figure S1. Detailed experimental setup for direct quantification of negative pressure generated by the microstructured suction-type patch.**

Figure S1 showing the experimental setup used for the direct quantification of negative pressure generated by the microstructured suction-type patch. Measurements were performed using a universal testing machine (UTM; Neo-Plus, Daejeon, Republic of Korea) equipped with a vertical loading stage.

A custom-fabricated flat sample holder was mounted on the UTM crosshead to ensure reproducible and mechanically stable application of normal load. The holder consisted of a rigid metallic square plate designed to securely fix the patch specimen in a horizontal orientation. The contact surface of the holder was planar and mechanically stiff, enabling uniform load transfer to the patch and minimizing bending or local deformation during testing. The microstructured suction-type patch was centrally mounted on the underside of the holder so that the patch–substrate interface remained aligned with the loading axis.

A skin-mimicking silicone elastomer substrate was fixed on the lower stage of the UTM. For the ampule-treated condition (CNP), the substrate surface was uniformly coated with the same functional ampule used in the clinical study prior to measurement. For the control condition, the substrate was used in a dry, untreated state.

During measurement, a constant normal load was applied via the UTM crosshead to bring the patch into contact with the substrate, simulating manual facial application conditions. Upon contact, the negative pressure generated at the patch–substrate interface by the microcavity structures was directly recorded using a calibrated pressure sensor connected to the interfacial region (sensor: Push-Pull Gauge HF-5; measurement max load: 50 N; resolution: 0.01N). The pressure signal was continuously monitored, and pressure values were collected at 0, 5, 10, and 15 min after initial contact (total application time: 15 min, consistent with the clinical protocol).

All measurements were conducted without any external vacuum pump, suction device, or active pressure control system, confirming that the recorded negative pressure originated solely from the passive suction effect of the microcavity architecture. This experimental configuration was used to obtain the time-dependent negative pressure profiles presented in Figure 2a, demonstrating sustained suction over a 15-min application period.

**
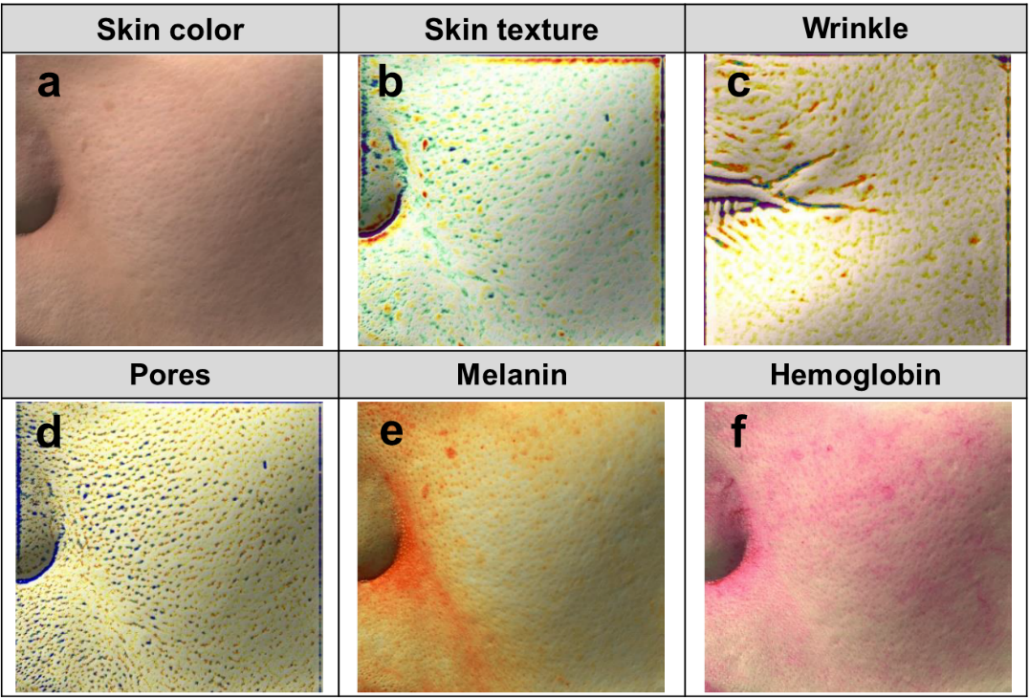
**

**Figure S2. Representative Antera 3D modality images:** (a) skin color, (b) skin texture, (c) wrinkle depth, (d) pore density, (e) melanin concentration, and (f) hemoglobin distribution. These six imaging modes allow for multiparametric visualization of skin conditions before and after patch application.

**Figure S2** demonstrates the multiparametric imaging capabilities of the Antera 3D® system, which was employed to assess various biophysical skin parameters before and after patch application. Skin color was captured as a baseline indicator of tone uniformity (Figure S2-a), while skin texture mapping highlighted microtopographic irregularities, including roughness and fine lines (Figure S2-b). Wrinkle depth was quantified through elevation-based color coding, providing a clear visual of relief patterns (Figure S2-c). Pore density and distribution were visualized to assess smoothness and oil control (Figure S2-d). Melanin and hemoglobin concentrations, shown in Figures S2-e and S2-f, respectively, allowed evaluation of pigmentation and superficial vascular conditions. Together, these six modalities offer a comprehensive overview of skin condition and enable objective, image-based comparisons across multiple aesthetic dimensions. Figure S2 illustrates how this integrated imaging approach supports holistic evaluation of treatment efficacy**.**

**
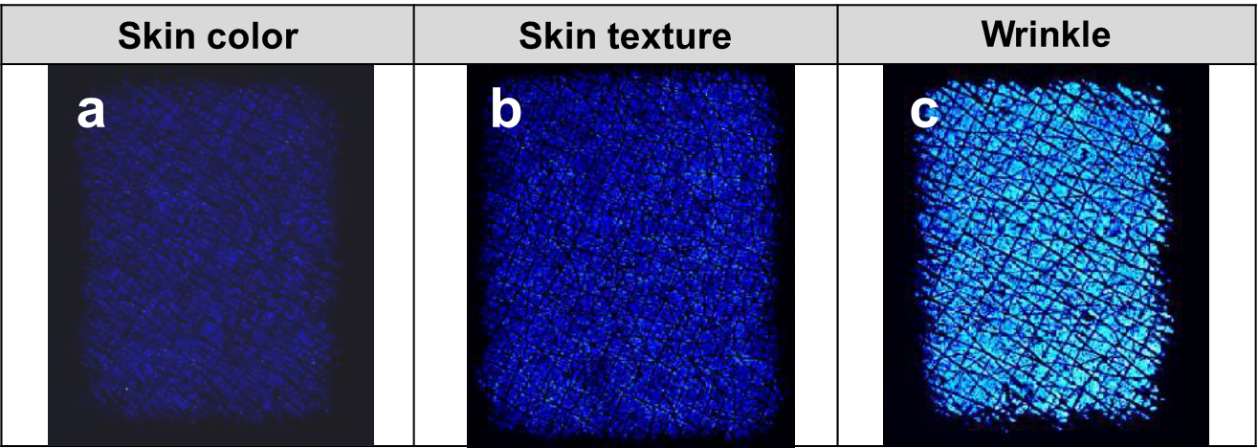
**

**Figure S3. Representative Epsilon E100 capacitance-based hydration imaging:** (a) skin color map, (b) skin texture map, and (c) wrinkle-associated dielectric map. Brighter areas correspond to higher dielectric constant (ε) values, indicating increased skin moisture content.

**Figure S3** presents representative Epsilon E100 imaging outputs that visualize skin hydration levels through capacitance mapping. As shown in Figure S3-a, baseline dielectric distribution was mapped across the skin, where darker regions indicated lower moisture content. Figure S3-b illustrates the microtexture of the skin, offering visual correlation between surface roughness and local hydration. In Figure S3-c, dielectric data were overlaid on wrinkle structures, revealing moisture variation along fine lines and deeper folds. Brighter areas in all images correspond to higher dielectric constant (ε) values, signifying increased skin moisture. These images in Figure S3 demonstrate the spatial resolution of E100-based capacitance imaging in capturing both the uniformity and depth of hydration, offering a quantitative and topographical assessment of moisturizing efficacy.

**
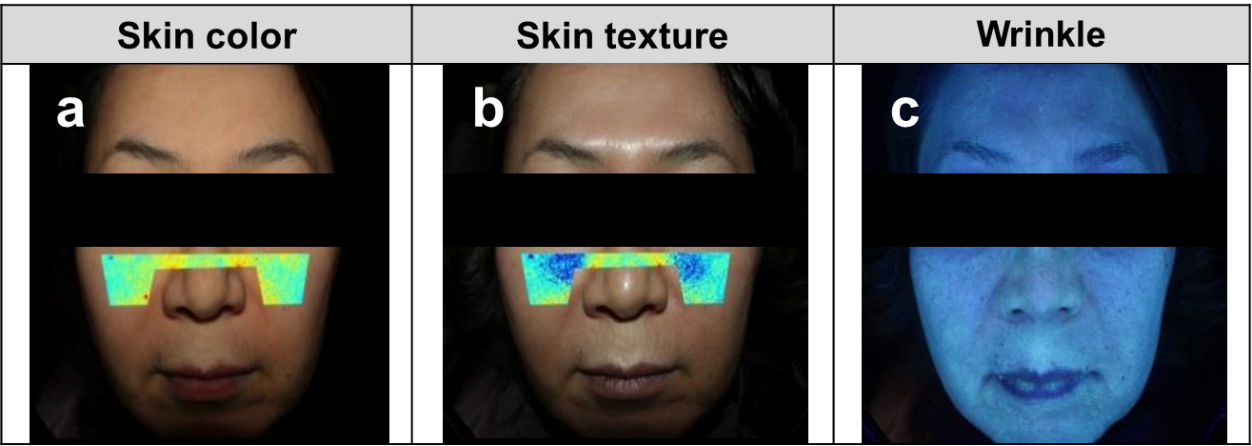
**

**Figure S4. DermaVision multi-modal pigmentation analysis:** (a) cross-polarized imaging (CPI) for superficial pigmentation, (b) parallel-polarized imaging (PPI) for texture-related melanin distribution, and (c) UV imaging for deep epidermal pigmentation assessment.

**Figure S4** illustrates the use of DermaVision’s multi-modal imaging system to assess skin pigmentation and tone uniformity across various optical depths. As shown in Figure S4-a, the cross-polarized imaging (CPI) mode captured superficial pigmentation at the stratum corneum, effectively highlighting surface-level tone unevenness. Parallel-polarized imaging (PPI), presented in Figure S4-b, visualized melanin patterns associated with textural features in the upper epidermis. Ultraviolet (UV) imaging (Figure S4-c) revealed deep epidermal pigmentation by detecting dermal melanin deposits that are typically undetectable under visible light. By combining these imaging modalities, the system provided a comprehensive view of pigmentation distribution across different skin layers, supporting quantitative and depth-resolved evaluation of patch-induced pigment modulation.

**REFERENCE**

1. Zare M, Ghomi ER, Venkatraman PD, Ramakrishna S. Silicone-based biomaterials for biomedical applications: Antimicrobial strategies and 3D printing technologies. *Journal of Applied Polymer Science.* 2021;138(38):50969.

2. Singh M, Teodorescu DL, Rowlett M, et al. A Tunable Soft Silicone Bioadhesive for Secure Anchoring of Diverse Medical Devices to Wet Biological Tissue. *Advanced Materials.* 2024;36(3):2307288.
